# Graphene/CNT Nanocomposites: Processing, Properties, and Applications

**DOI:** 10.3390/nano16020100

**Published:** 2026-01-12

**Authors:** Sachin Kumar Sharma, Slavica Miladinović, Lokesh Kumar Sharma, Sandra Gajević, Yogesh Sharma, Mohit Sharma, Stefan Čukić, Blaža Stojanović

**Affiliations:** 1 Surface Science and Tribology Lab, Department of Mechanical Engineering, Shiv Nadar Institution of Eminence, Gautam Buddha Nagar, Greater Noida 201314, India; 2 University of Kragujevac, Faculty of Engineering, Sestre Janjić 6, 34000 Kragujevac, Serbia; slavicam@kg.ac.rs (S.M.); stefan.cukic@kg.ac.rs (S.Č.); blaza@kg.ac.rs (B.S.); 3 Department of Physics, GLA University, Mathura 281406, India; 4 Department of Physics & Environmental Sciences, Sharda School of Engineering & Science, Sharda University, Greater Noida 201310, India; uvsbhu@gmail.com; 5 Department of Physics and Material Science, Jaypee University, Anoopshahr 203390, India; mohit.sharma@mail.jaypeeu.ac.in

**Keywords:** graphene, carbon nanotubes (CNTs), processing, metal and ceramic nanocomposites, interface engineering

## Abstract

Carbon nanotube (CNT) and graphene-reinforced nanocomposites have become exceptional multifunctional materials because of their exceptional mechanical, thermal, and electrical properties. Recent developments in synthesis methods, dispersion strategies, and interfacial engineering have effectively overcome agglomeration-related limitations by significantly improving filler distribution, matrix compatibility, and load-transfer efficiency. These nanocomposites have better wear durability, corrosion resistance, and surface properties like super-hydrophobicity. A comparative analysis of polymer, metal, and ceramic matrices finds benefits for applications in biomedical, construction, energy, defense, and aeronautics. Functionally graded architecture, energy-harvesting nanogenerators, and additive manufacturing are some of the new fabrication processes that enhance design flexibility and functional integration. In recent years, scalability, life-cycle evaluation, and environmentally friendly processing have all gained increased attention. The development of next-generation, high-performance graphene and carbon nanotube (CNT)-based nanocomposites is critically reviewed in this work, along with significant obstacles and potential next steps.

## 1. Introduction

The development of nanocomposite materials, made possible by advances in nanotechnology and nanoscale characterization techniques, represents a significant turning point in the history of contemporary materials engineering [1]. Multi-phase materials known as nanocomposites, in which at least one phase has dimensions in the nanometer scale (usually less than 100 nm), have demonstrated the ability to significantly change bulk properties by customized manipulation of interfaces and microstructures [2]. Carbon-based nanomaterials, particularly graphene and carbon nanotubes (CNTs), have shown unmatched potential in reinforcing traditional matrices including polymers, metals, and ceramics among the wide range of nanofillers studied [3]. The exceptional intrinsic properties of these two-dimensional (2D) and one-dimensional (1D) nanostructures outperform those of conventional reinforcements. With a honeycomb lattice and a single layer of sp^2^ hybridized carbon atoms, graphene exhibits remarkable mechanical strength (Young’s modulus > 1 TPa), electrical conductivity (~106–108 S/m), and thermal conductivity (3000–5000 W/mK) [4]. Cylindrical graphene derivatives, or CNTs, have ballistic electron transport capabilities, tremendous flexibility, and ultrahigh tensile strengths (>100 GPa) [5]. These nanostructures produce materials with superior multifunctionality when successfully integrated into bulk matrices, creating new opportunities in flexible electronics, medicinal devices, aerospace, and energy storage [6]. Even with their inherent benefits, graphene and carbon nanotube-based nanocomposites still face significant obstacles in achieving their theoretical promise in real-world applications. The dispersion state, alignment, interfacial adhesion, and volume fraction of these reinforcements inside the host matrix are intrinsically linked [7]. Agglomeration and poor matrix wettability are caused by high π–π interactions and van der Waals forces in pristine graphene and CNTs [8]. Electrical percolation networks weaken, stress transfer is impeded, and homogeneity is limited. Consequently, a great deal of research has been done on creating efficient dispersion techniques that maintain the integrity of nanocarbons while enhancing matrix compatibility. These techniques can be covalent (functional group grafting, oxidation, diazonium modification) or non-covalent (π–π stacking, polymer wrapping, surfactant-assisted dispersion) [9]. Techniques for hybridization, in which graphene/CNTs are combined with secondary reinforcements like metal nanoparticles or ceramic nano-inclusions, have demonstrated promise in balancing mechanical stiffness, electrical conductivity, and thermal stability in a range of working conditions [10].

Graphene and carbon nanotubes (CNTs) perform as structural and functional reinforcements in polymer matrix nanocomposites (PMNCs), significantly improving mechanical properties, thermal stability, and conductivity even at low filler loadings (<5 wt.%) [11]. Thermosets (like epoxy and phenolic resin) and thermoplastics (PE (Polyethylene), PS (Polystyrene), and PEEK (Polyether ether ketone)) have been effectively reinforced to produce notable improvements in fracture toughness, thermal deflection temperature, and Young’s modulus [12]. Furthermore, the high modulus and strength of nanocarbons are complemented by the natural flexibility of polymers, resulting in high-performance lightweight materials [13]. Functionalized carbon nanotubes in epoxy matrices increased electrical conductivity and tensile strength by as much as 60% [14,15]. Furthermore, hybrid PMNCs that combine graphene and carbon nanotubes with oxide nanofillers like ZnO and TiO_2_ have demonstrated improved electromagnetic and photocatalytic qualities for smart coatings and sensors [16]. Graphene and carbon nanotube (CNT) reinforced metal matrix nanocomposites (MMNCs) have also drawn a lot of interest, particularly for structural and aerospace applications that require high strength-to-weight ratios and elevated thermal conductivity [17]. When reinforced with carbon nanostructures, metals like aluminum, magnesium, copper, and titanium show significant increases in yield strength, microhardness, and thermal stability [18]. To maintain nanostructure shape, avoid interfacial degradation, and guarantee uniform distribution, processing methods including powder metallurgy, friction stir processing, spark plasma sintering (SPS), and additive manufacturing (AM) have been refined [19]. To prevent carbide production and matrix embrittlement, it is crucial to manage the processing temperature and the sintering environment [20,21]. The synergistic strengthening processes of MMNCs, which are enhanced by nanoscale dispersion and chemical interaction between reinforcements and matrix, include Orowan looping, grain boundary pinning, and load transfer via interfacial shear stress [22].

The use of graphene and carbon nanotubes (CNTs) has greatly improved ceramic matrix nanocomposites (CMNCs), despite their historical limitations due to brittleness and processing complexity [23]. Crack bridging, pull-out, and intergranular stress redistribution are some of the mechanisms that give alumina, silicon carbide, and zirconia-based matrices greater fracture toughness, wear resistance, and thermal shock stability [24]. The preservation of nanocarbon structural integrity during high-temperature sintering processes is the difficulty in CMNCs [25]. Dense, consistent CMNCs have been successfully created using cutting-edge methods such chemical vapor infiltration (CVI) and spark plasma sintering [26]. Because ceramic-based nanocomposites dissipate energy more effectively during crack propagation, they can increase toughness by up to 100% when compared to monolithic ceramics, especially in graphene platelet-reinforced alumina composites [27,28]. Additionally, the electrical conductivity that nanocarbons impart enables ceramics to be used in a variety of multifunctional applications, such as energy storage devices, sensors, and actuators [29]. Applications for graphene and carbon nanotube-reinforced nanocomposites are not just structural. Super-hydrophobicity, corrosion resistance, EMI shielding, self-sensing, thermal management, and even energy harvesting are just a few of the many functions made possible by their special qualities [30]. By creating winding diffusion channels that prevent moisture and ion infiltration, graphene improves barrier qualities in coatings [31,32]. Graphene and carbon nanotubes (CNTs) are solid lubricants that reduce wear and friction in dry sliding environments by forming in situ transfer layers [33]. Such tribological nanocomposites have been investigated for use in automotive components and aircraft bearing systems [34,35]. Furthermore, developments in direct ink writing and 3D printing have made it possible to create anisotropic architectural nanocomposites with gradient qualities that can be used in soft robotics, biomedical implants, and aerospace [36].

A strategy change toward utilizing the synergistic effects of several nano-reinforcements is shown in increasing interest in multiphase and hybrid nanocomposites. CNTs and graphene combined in a polymer matrix, for example, have been shown to improve network connection, lower percolation thresholds, and increase mechanical flexibility [37]. By adding specific functions (such as antibacterial behavior, magnetism, and dielectric response), metallic or ceramic nanoparticles further enhance this design [38]. Functional gradient nanocomposites (FGNs) provide mechanical anisotropy and spatially tailored performance due to the spatial variation in filler concentration [39]. New research indicates that filler ratios, dispersion characteristics, and process conditions may be predicted using machine learning (ML) and computer modeling, which will speed up materials discovery and minimize experimental trial-and-error [40]. Graphene and carbon nanotube (CNT)-based nanocomposites are becoming more sustainable due to environmental concerns and regulatory frameworks [41]. Low-energy processing methods, biodegradability, recyclability, and life cycle analysis (LCA) are increasingly important when choosing materials. Nanocarbon-reinforced biodegradable polymer matrices are being developed for disposable sensors and temporary electronics [42]. Additionally, research is being done on the biocompatibility of functionalized graphene and carbon nanotubes (CNTs) for use in tissue engineering, drug administration, and bone scaffolds. It has been suggested that cytotoxicity can be reduced by carefully regulated surface chemistry [43].

The review showed shifting from descriptive overview of graphene/CNT nanocomposites to a mechanism-driven synthesis that unifies structure–property relationships across polymer, metal, and ceramic matrices. Unlike existing reviews that predominantly treat these systems separately, this work identifies common governing parameters namely dispersion quality, interfacial load-transfer efficiency, percolation behavior, and processing-induced size effects and demonstrates their universal role in controlling mechanical and functional performance. Importantly, the review integrates representative experimental evidence, including stress–strain responses, filler-loading trends, and conductivity evolution, to quantitatively validate these mechanisms rather than relying solely on schematic interpretation. By correlating processing routes with microstructural evolution and macroscopic properties, the review provides predictive insight into hybrid graphene/CNT architectures. This unified, experimentally grounded framework enhances reproducibility, supports rational material design, and facilitates the translation of graphene/CNT nanocomposites from laboratory-scale studies to application-ready engineering materials.

## 2. Recent Advances and Emerging Trends

Research on the development and diversification of carbon-based nanocomposites, especially those reinforced with graphene and carbon nanotubes (CNTs), has increased at an unparalleled rate in recent years. Because these materials may concurrently impart strength, electrical conductivity, thermal stability, and multifunctionality to a wide range of matrices, including polymers, metals, and ceramics, they continue to pique the curiosity of both academics and industry. However, overcoming enduring obstacles in dispersion, alignment, interface engineering, and scalable production is necessary to fully realize the potential of these nanofillers. As a result, new approaches, cutting-edge processing techniques, and hybrid design are changing the course of nanocomposite development going forward. The introduction of multiscale hybridization and 3D hierarchical architecture has been one of the most important developments in this field [44]. Modern methods concentrate on creating three-dimensional, interconnected networks of graphene and CNTs within the host matrix, in contrast to conventional nanocomposites where nanofillers are distributed as separate phases. These networks serve as constant channels for heat conductivity, electron transport, and mechanical load transmission [45]. For example, graphene aerogels and CNT scaffolds with adjustable porosity and anisotropic characteristics have been created using freeze-drying, hydrothermal assembly, and directed freezing approaches. These scaffolds maintain structural continuity when infiltrated with metals or polymers, allowing for high-performance composites with percolated filler networks that greatly improve mechanical integrity and conductivity.

Hybrid nanofiller systems are increasingly popular in conjunction with 3D structuring to cooperatively utilize the complementing qualities of various nanostructures. For example, a bridging and sheeting effect that improves interfacial connectivity and lowers the percolation threshold can result from mixing graphene (with its 2D sheet shape and outstanding barrier properties) with CNTs (which give 1D reinforcement and high aspect ratio) [46]. Furthermore, ternary nanocomposites with extra nanoparticles like magnetic (FeO_4_), ceramic (AlO_3_, TiO_2_), or metallic (Ag, Au) fillers are being intensively investigated. These multipurpose systems pave the way for thermoelectric applications, self-sensing materials, and customized electromagnetic interference (EMI) shielding [47]. Controlled surface functionalization and self-assembly methods that guarantee close contact and uniform dispersion within the matrix frequently make the hybridization approach possible. The use of machine learning (ML) and data-driven modeling in the design and optimization of nanocomposites is another development with broad ramifications. Computational technologies that can forecast structure–property interactions are rapidly replacing or supplementing traditional trial-and-error experimental methods. Recent advances increasingly leverage modeling and data-driven approaches to predict dispersion quality, percolation thresholds, and interfacial stress evolution in hybrid graphene/CNT systems [48]. Such frameworks enable rational design of nanocarbon building-block architectures by integrating size-dependent electronic structure and network connectivity into materials optimization strategies. To find the best combinations of matrix, nanofiller type, functionalization, volume fraction, and processing route for specific applications, supervised learning algorithms are being trained on sizable datasets derived from experimental and simulated results [49]. In nanocomposites, ML frameworks have demonstrated promise in estimating mechanical strength, conductivity, thermal diffusivity, and damage evolution. These technologies enable faster discovery and customization by drastically lowering design cycles, costs, and experimental burden.

Another revolutionary advancement in the processing of nanocomposite materials is additive manufacturing (AM), sometimes known as 3D printing. It became more sophisticated due to the viability of creating graphene- and carbon nanotube-based nanocomposites by extrusion-based methods including fused deposition modeling (FDM), direct ink writing (DIW), and stereolithography (SLA) [50]. Creating printable inks and filaments with the right rheological, thermal, and mechanical characteristics is essential to effective AM. Critical criteria include shear-thinning behavior, appropriate filler dispersion, and post-processing procedures like thermal annealing or photopolymerization [51]. Functionally graded materials and bespoke geometries for biomedical implants, flexible electronics, and lightweight aerospace components are made possible by additive manufacturing’s unparalleled control over the microstructure, anisotropy, and spatial distribution of fillers [52]. The development of bio-inspired and stimuli-responsive interfaces has opened new possibilities for adaptive and self-healing nanocomposites in the field of interfacial engineering. Researchers are creating hierarchical interphases with reversible bonding, energy dissipation, and toughness, drawing inspiration from natural systems like nacre and spider silk [53]. For example, dynamic covalent chemistry can create chemical or thermally reversible networks at the filler–matrix interface using disulfide bonds or Diels-Alder processes. This results in recyclability, damage tolerance, and self-healing activity [54]. Additionally, by incorporating thermo-responsive and mechano-responsive moieties into the interfacial region, smart materials that can change their electrical or mechanical characteristics in response to external stimuli are made possible, opening exciting possibilities for soft robotics, wearable electronics, and artificial muscles [55].

**Figure 1:**
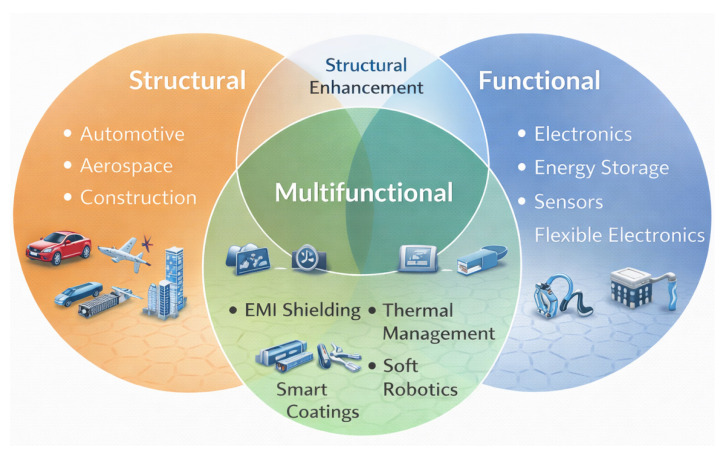
Application landscape of graphene/CNT nanocomposites, illustrating the classification of structural, functional, and multifunctional applications and their overlap, highlighting the versatility of hybrid nanocarbon systems across sectors such as automotive, aerospace, electronics, energy storage, EMI shielding, thermal management, and smart coatings.

Conventional acid oxidation has given way to more sophisticated and selective chemistry in surface functionalization processes at the same time. More controlled grafting of functional groups is possible via click chemistry, diazonium coupling, and salinization without sacrificing the structural integrity of graphene or carbon nanotubes [56]. Furthermore, reversible and non-destructive functionalization is made possible by supramolecular techniques like hydrogen bonding and host-guest interactions. To customize the nanofiller surface properties, recent research has also looked into microwave-assisted changes, plasma treatment, and electro polymerization [57]. Stronger interfacial bonding, improved dispersion, and programmable interfacial energy are made possible by these methods, all of which are essential for enhancing the performance and dependability of nanocomposites in service contexts. Green synthesis, biocompatibility, and recyclability of carbon nanocomposites are becoming increasingly important from an environmental and sustainability standpoint, according to new research. Many studies are being conducted on the use of bio-based polymers as matrices, including cellulose derivatives, chitosan, and polylactic acid (PLA) [58]. Concurrently, energy-intensive techniques and dangerous solvents are being replaced by environmentally benign processing pathways that use solvent-free melt mixing, supercritical fluids, and water-based dispersions [59]. Furthermore, methods for recovering and reusing graphene or carbon nanotubes from end-of-life items are becoming more popular. To recover nanofillers for circular material economy, electrochemical delamination, thermal degradation, and selective dissolution are being developed [60]. Particularly in the automotive and electronic industries, life-cycle assessments (LCAs) are being used to study the environmental impact of nanocomposite deployment and manufacture. The use of carbon nanocomposites in developing energy technology, specifically in flexible batteries, supercapacitors, and thermoelectric devices, is another quickly developing field [61]. Because of their high electrical conductivity, huge surface area, and adjustable electrochemical characteristics, graphene and carbon nanotubes (CNTs) are excellent options for electrode materials providing flexible, lightweight, and robust films that can withstand mechanical deformation without experiencing a decline in performance when integrated into polymer matrices. Furthermore, to improve charge transport, ion diffusion, and electrode–electrolyte interactions, sophisticated topologies such sandwich structures, core–shell configurations, and multilayered composites are being developed. Higher thermoelectric figure of merit (ZT) values result from the addition of graphene or carbon nanotubes (CNTs), which enhance the Seebeck coefficient and decrease heat conductivity [62]. These developments are essential to the creation of wearable, portable, and self-sufficient energy systems. The expanding application landscape of graphene/CNT nanocomposites, spanning structural, functional, and multifunctional domains, is summarized schematically in Figure 1.

Recent developments in the field of sensing and actuation include the application of carbon nanocomposites for triboelectric, capacitive, and piezoresistive sensors. Percolative networks that show notable resistance variations under strain or pressure can be created thanks to the high aspect ratio and conductivity of CNTs and graphene [63]. These sensors are being used in human–machine interactions, electronic skin (e-skin), and structural health monitoring. Sensor arrays can now be printed on stretchy and flexible substrates because of developments in ink composition and substrate compatibility. Additionally, the integration of these sensors into biomedical patches, gloves, or textiles allows for the real-time monitoring of physiological signals including mobility, respiration, and heartbeat [64]. The use of graphene and CNT-based composites is being driven by the need for lightweight, corrosion-resistant, and thermally stable materials in electronic packaging and EMI shielding [65]. Polymer-based alternatives with tailored nanofiller networks are replacing traditional metal-based solutions. Due to numerous reflection, absorption, and conductivity losses, studies have shown that shielding effectiveness in thin composite materials exceeds 60–80 dB [66]. To further improve shielding efficacy, new architecture is being developed, such as aligned fillers, multilayered structures, and hybrid foams. Furthermore, dual-mode shielding (electric and magnetic) is made possible by the addition of magnetic nanoparticles to carbon nanostructures, expanding the spectrum of frequencies and environmental conditions that can be used.

## 3. Graphene and CNT-Reinforced Nanocomposites Across Different Matrices

In the realm of advanced materials, the creation of nanocomposites utilizing carbon-based nanostructures like graphene and carbon nanotubes (CNTs) signifies a paradigm change. These nanocomposites can overcome the constraints of conventional reinforcement in metals, ceramics, and polymers because of their exceptional mechanical, thermal, and electrical properties [67]. The previous research on graphene and carbon nanotube-reinforced nanocomposites is critically summarized in this section, with an emphasis on matrix-specific difficulties, dispersion techniques, interfacial interactions, and performance results. Reviewing the methods used to get around obstacles in filler–matrix compatibility, fabrication procedures, and functional optimization is emphasized.

### 3.1. Graphene and CNTs in Polymer Nanocomposites

Polymers, with their low processing temperatures, tunable physical properties, lightweight nature, and ease of functionalization, have consistently emerged as the most versatile and industrially relevant platforms for the integration of graphene and carbon nanotubes (CNTs) among the various matrices explored for nanocomposite development [68,69]. Thermosetting resins like epoxy and vinyl ester, as well as thermoplastic polymers like polyethylene (PE), polystyrene (PS), and polypropylene (PP), have both been thoroughly investigated for nanofiller insertion [70]. These polymers function as efficient host matrices that can convert the inherent characteristics of graphene and carbon nanotubes into improvements in macro-scale performance. By examining polymer nanocomposites reinforced with CNTs and graphene derivatives, Mittal et al. provided a thorough foundation [71]. The results show that even with relatively low filler loadings, ranging from 0.1 wt.% to 2.0 wt.%, notable gains in tensile strength, Young’s modulus, and impact resistance can be made. This is due to the nanofillers’ remarkable mechanical qualities, high aspect ratio, and surface area, which, when evenly distributed throughout the polymer matrix, enable effective stress transfer. It has been shown that graphene nanoplatelets (GNPs) can significantly improve heat conductivity in polymeric systems [72]. Kumar et al. found that, in epoxy/GNP systems, thermal conductivity can rise to 30 times above the baseline, if there is robust interfacial contact and homogeneous dispersion [73]. However, the strong π–π interactions between graphene sheets and the tendency of CNTs to entangle make it difficult to achieve such uniform dispersion [74]. Several functionalization strategies have been used to get around this. A methodical categorization of dispersion and functionalization techniques specifically designed for CNTs was presented by Ma et al. [75]. These include non-covalent methods like solution blending and surfactant-assisted exfoliation, as well as covalent functionalization, which involves grafting chemical groups onto the nanotube surface to increase solubility and interfacial adhesion. These methods are essential for regulating filler distribution, encouraging interactions between polymers and nanofillers, and preserving the fillers’ structural integrity.

**Figure 2:**
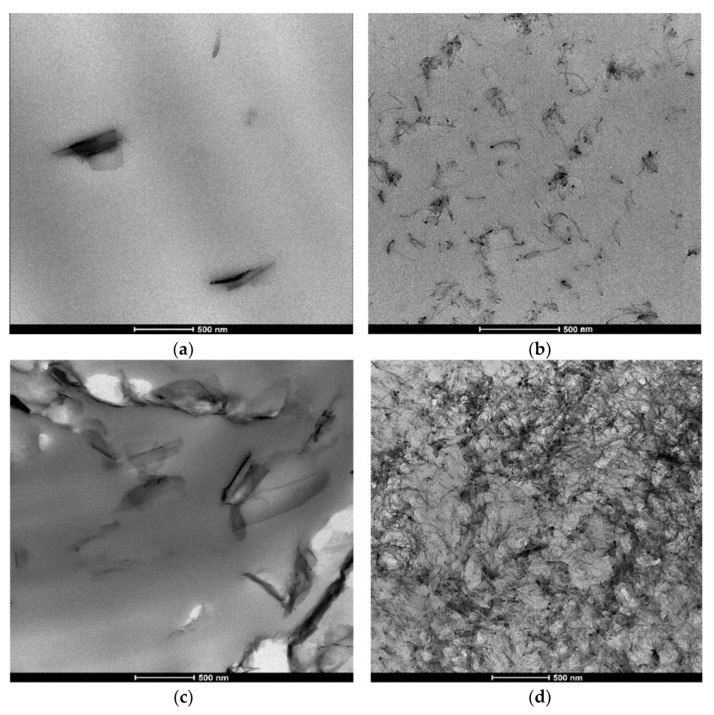
TEM micrographs of PLA-based nanocomposites with varying filler type and content: (**a**) 1.5 wt.% GNP/PLA, (**b**) 1.5 wt.% MWCNT/PLA, (**c**) 9 wt.% GNP/PLA, and (**d**) 9 wt.% MWCNT/PLA, all captured at identical magnification [76].

Hybrid reinforcing techniques that combine 1D (CNTs) and 2D (graphene) nanofillers have demonstrated better multifunctional performance in recent advancements. Modeled as yield-stress fluids, Kotsilkova and Tabakova’s [76] PLA nanocomposites filled with GNP and MWCNT demonstrate significant shear-thinning and flow instabilities, with rheological characteristics connecting filler structure to printability and multifunctionality. TEM micrographs of GNP/PLA and MWCNT/PLA composites with 1.5 and 9 wt.% fillers are shown in Figure 2a–d. Based on electrical and rheological research, the findings are consistent with previously determined percolation thresholds of ≥1.5 wt.% for MWCNTs and ≥3 wt.% for GNPs in PLA. Such GNP-CNT hybrid systems were investigated by Abedi et al., who discovered that the fillers’ synergistic interactions create interconnected percolation networks [77]. In comparison to single-filler composites, these networks not only improve mechanical characteristics and fracture toughness but also greatly increase the effectiveness of electromagnetic interference (EMI) shielding by more than 50%. These hybrid structures provide enhanced load transfer and conductive pathways by utilizing the special geometrical and electrical synergies between graphene and carbon nanotubes [78,79]. Furthermore, graphene is very appealing for specialized applications such barrier packaging, corrosion resistance, and smart electronics because of its intrinsic qualities, such as strong electrical conductivity, wide surface area, and impermeability to gases [80]. Razack et al. investigated its application in cutting-edge fields such as tribological coatings and stretchable electronics [81]. In addition to improving structural toughness, graphene-based fillers make it possible for self-lubricating surfaces to develop, which lowers friction and wear and opens new possibilities for dynamic and wearable material systems [82]. All things considered, advancements in filler processing, interface engineering, and hybrid design techniques are propelling the continued evolution of polymer-based nanocomposites including graphene and carbon nanotubes. These advancements highlight how these systems could be used to satisfy the expanding need for multipurpose materials in the medicinal, electronics, automotive, and aerospace industries.

### 3.2. Carbon-Based Nanofillers in Metal Matrix Composites

Due to the urgent need for advanced materials that provide remarkable mechanical strength, lightweight structure, thermal conductivity, and multifunctional performance, the incorporation of carbon-based nanofillers, such as graphene and carbon nanotubes (CNTs), into metal matrices has attracted more attention in the last ten years [83]. In the aerospace, automotive, electronics, and military industries, metal matrix nanocomposites (MMNCs) reinforced with graphene and carbon nanotubes (CNTs) have shown promise as structural and functional components [84]. Due to their favorable mechanical characteristics and compatibility with carbon nanostructures, aluminum (Al), magnesium (Mg), copper (Cu), and titanium (Ti) stand out among the many metal matrices examined [85]. According to Tjong et al.’s evaluation of the oldest and most thorough research on the topic, adding carbon nanostructures can result in appreciable gains in yield strength, hardness, fracture toughness, and thermal stability [86]. However, several complex parameters, including matrix–filler compatibility, interfacial bonding, nanofiller dispersion, and the thermal/mechanical processing conditions employed during fabrication, affect how well graphene and CNTs reinforce within metallic matrices [87,88].

Getting nanofillers to evenly disperse throughout the metal matrix without sacrificing their inherent structural integrity is one of the main challenges in creating MMNCs [89]. Spark plasma sintering (SPS) and powder metallurgy have emerged as the most successful advanced processing techniques developed to address this issue [90]. With these methods, the excessive heat breakdown of carbon nanomaterials can be reduced, and microstructural development can be precisely controlled. Rapid densification at relatively low temperatures is an advantage of SPS, which maintains the morphology of the nano-fillers and guarantees efficient load transmission between the reinforcement and matrix [91]. In copper- and aluminum-based nanocomposites, spark plasma sintering (SPS) has proven particularly effective in translating nanoscale reinforcement into macroscopic property enhancement [92]. For instance, graphene- and CNT-reinforced Cu systems consolidated by SPS exhibit significant grain refinement, with reported crystallite sizes reduced to the 20–40 nm range. This refinement directly contributes to improved yield strength and hardness through Hall–Petch strengthening and grain boundary pinning [93]. The rapid heating and short dwell times characteristic of SPS minimize interfacial reactions and preserve nanocarbon integrity, resulting in high relative densities (typically 95–97%) and enhanced load transfer efficiency [94]. These observations highlight the critical role of size effects and consolidation kinetics in determining the mechanical performance of metal matrix nanocomposites.

Several dispersion techniques, such as solution-based molecular mixing, ultrasonication, and high-energy ball milling, were assessed by Baig et al. [95]. When the ball-to-powder ratio, rotation speed, and milling time are adjusted, high-energy ball milling has shown the best results in creating consistent nanofiller dispersion among these [96]. Ball milling can be precisely adjusted to balance dispersion uniformity and filler integrity, even if shear and impact pressures may cause some structural damage to graphene and carbon nanotubes [97]. Additionally, CNT bundles have been disentangled and graphene layers exfoliated by ultrasonication in appropriate solvents before mechanical mixing [98].

To improve CNTs’ and graphene’s compatibility with metallic matrices, surface functionalization is essential. Wetting at the filler–matrix interface, preventing agglomeration, and improving dispersion stability can all be achieved through covalent or non-covalent functionalization [99]. To improve metallurgical bonding, CNTs can be decorated with metallic ions like nickel (Ni) or silver (Ag) [100]. In comparison to their uncoated counterparts, aluminum matrix composites containing Ni-coated carbon nanotubes (CNTs) showed a remarkable 40% increase in ultimate tensile strength, according to Song et al. [101]. This increase was attributed to enhanced interfacial load transfer and the activation of dislocation generation mechanisms during deformation. In addition, the combined application of hybrid reinforcements made of ceramic and graphene nanoparticles has demonstrated promise in enhancing corrosion resistance, tribological strength, and mechanical strength. Graphene has been hybridized with ceramics like alumina (Al_2_O_3_), silicon carbide (SiC), and titanium diboride (TiB_2_) to create materials with improved thermal shock resistance, reduced friction coefficients, and higher wear resistance [102]. These enhancements result from graphene’s capacity to offer a lubricating layer while ceramic particles serve as load-bearing phases and obstacles to the spread of cracks. The creation of next-generation MMNCs can be approached in a variety of ways by incorporating such hybrid systems into metal matrices [103]. In metal matrix systems, the application of carbon-based nanofillers has great promise for customizing mechanical, thermal, and functional characteristics. However, important issues including thermal stability, scalable dispersion, and interfacial engineering are still being researched. The full potential of graphene and carbon nanotube-reinforced MMNCs for high-performance engineering applications is anticipated to be unlocked by further advancements in fabrication techniques and surface chemical modification.

### 3.3. Ceramic Matrix Nanocomposites Reinforced with Graphene and CNTs

**Figure 3:**
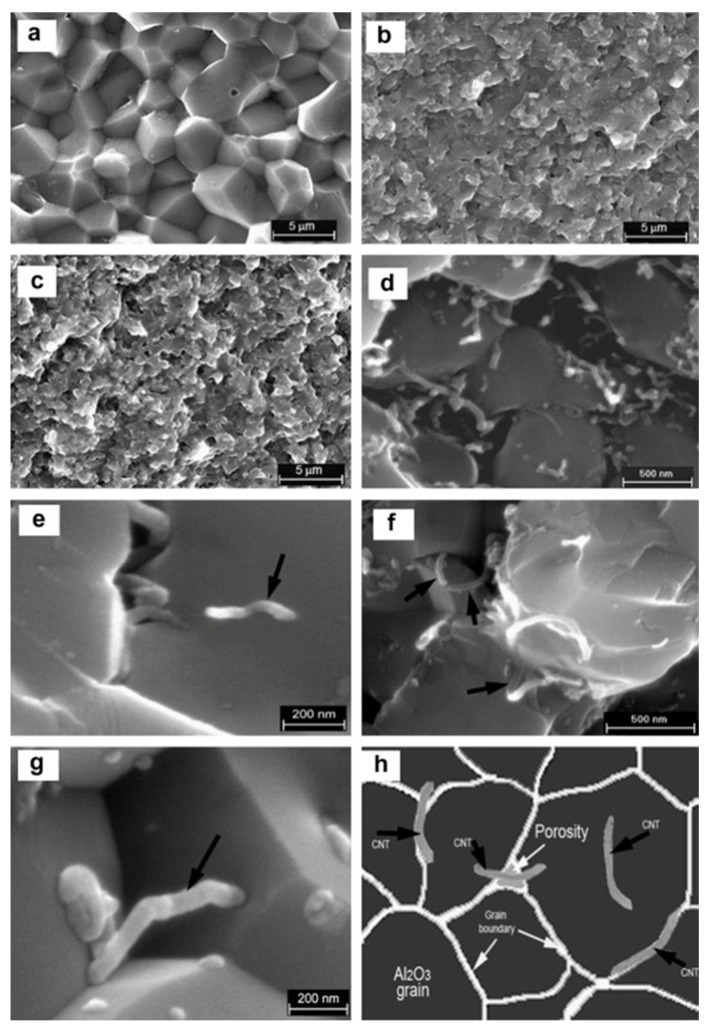
SEM images of fracture surfaces of (**a**) pure Al_2_O_3_, (**b**) Al_2_O_3_–2 wt.% CNTs, and (**c**) Al_2_O_3_–5 wt.% CNTs. (**d**) Higher-magnification image showing CNT dispersion within the matrix. Black arrows in (**e**) indicate CNTs embedded within Al_2_O_3_ grains, in (**f**) CNTs located at grain boundaries, and in (**g**) CNTs bridging adjacent grains, illustrating intragranular reinforcement, grain-boundary strengthening, and crack-bridging mechanisms, respectively. (**h**) Schematic summarizing typical CNT locations and associated toughening mechanisms in the Al_2_O_3_ matrix [106].

In engineering applications where high hardness, wear resistance, and chemical stability are required, ceramic materials are frequently utilized [104]. However, their structural performance is severely limited by their inherent brittleness and low damage tolerance, particularly when subjected to mechanical or thermal shock loading. By improving fracture toughness, flexural strength, thermal stability, and tribological performance, the addition of nanoscale carbon reinforcements—specifically, carbon nanotubes (CNTs) and graphene—to ceramic matrices is a viable way to get beyond these restrictions [105]. Because of their remarkable tensile strength and Young’s modulus, carbon nanostructures incorporated in ceramics like alumina (Al_2_O_3_), silicon carbide (SiC), and zirconia (ZrO_2_) function as efficient fracture arresters and load distributors. Ahmad et al. [106] prepared using hot pressing MWCNT-reinforced AlO_3_ nanocomposites. Significant improvements in hardness, flexural strength, and fracture toughness were noted at 2 wt.% CNTs. Because of difficulties with densification and dispersion, 5 wt.% CNTs marginally decreased hardness and strength while increasing toughness. The creation of an interfacial Al_2_O phase through carbothermal reduction, which guaranteed robust bonding, efficient load transmission, and crack-bridging and improved mechanical performance, was the primary driver of toughness enhancement. The morphology and fracture properties of different alumina (AlO_3_) composite systems are depicted in the SEM micrographs. The broken surface of pure monolithic Al_2_O_3_ is shown in Figure 3a, demonstrating its distinctive brittle failure with little plastic deformation. The cracked surface of AlO_3_ reinforced with 2 wt.% carbon nanotubes (CNTs) is depicted in Figure 3b. The early signs of CNT reinforcement effects, like enhanced crack deflection and grain bridging, are visible. The Al_2_O_3_ composite with 5 wt.% of CNTs, on the other hand, shows a more noticeable toughening effect in Figure 3c, which is explained by the CNTs’ improved dispersion and interaction within the matrix. With black arrows pointing to the exact locations of CNTs, a higher magnification SEM image in Figure 3d shows the detailed distribution of CNTs within the AlO_3_ matrix. This micrograph demonstrates the orientation and embedding of CNTs in different areas of the composite. Figure 3e shows CNTs inside the alumina grains, which may help with intragranular strengthening, and Figure 3f shows CNTs at the grain boundaries, which may serve as barriers to fracture propagation or grain boundary sliding. Additionally, Figure 3g shows instances in which CNTs operate as bridge components between neighboring grains, preventing crack propagation and improving the composite’s fracture toughness. The usual and advantageous locations of CNTs (shown by black arrows) within the AlO_3_ matrix, including their occurrence inside grains, at grain interfaces, and bridging across microstructural features, are conceptually summarized in the schematic diagram Figure 3h. By occupying key microstructural positions, CNTs enhance the overall mechanical performance of the nanocomposite, as this schematic demonstrates. According to Liu et al., CNTs increase fracture energy and postpone catastrophic failure in ceramic matrices by introducing toughening mechanisms such crack bridging, crack de-flection, and pull-out [107]. Because of its two-dimensional planar structure and high aspect ratio, graphene suppresses microcrack coalescence at high temperatures, hinders grain boundary migration, and improves interfacial load transfer [108]. To promote fine-grained microstructures, which are known to have better hardness and wear resistance, graphene and carbon nanotubes (CNTs) can also inhibit the formation of sintering grains [109]. In comparison to unreinforced alumina, Sharma et al. showed that hybrid reinforcements of graphene nano-platelets (GNPs) and carbon nanotubes (CNTs) in alumina matrices produce a twofold increase in hardness and a 70% decrease in wear rate [110]. Both the intrinsic lubricating qualities of carbon nanoparticles under tribological stress and the mechanical interlocking of fillers with ceramic grains are responsible for these improvements.

**Figure 4:**
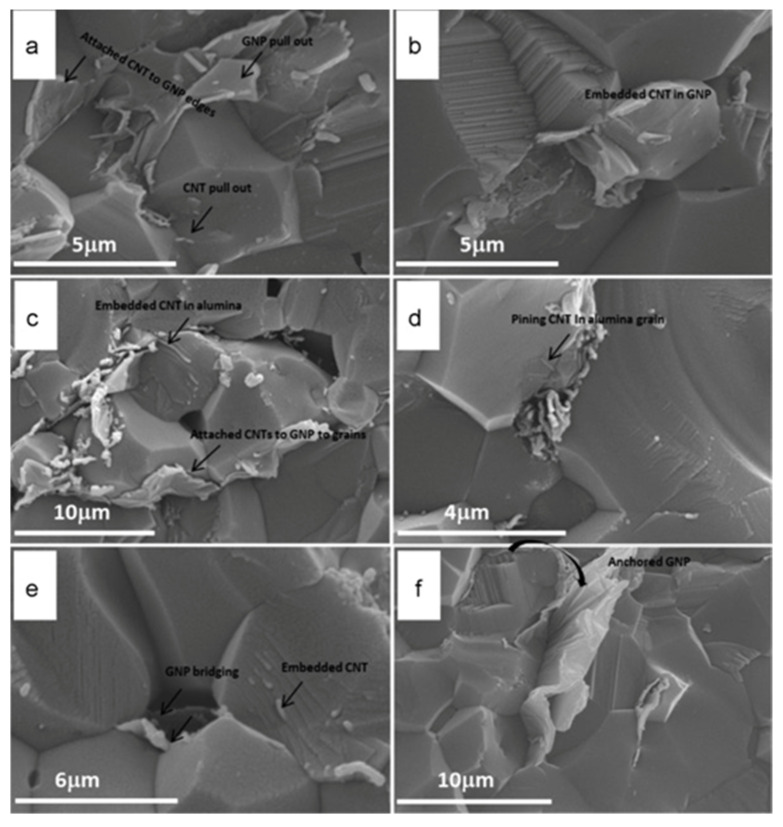
SEM images of fractured surfaces of Al_2_O_3_/GNP-CNT nanocomposites where GNP and CNT weight fraction varied as (**a**,**b**) 0.5 wt.% and 1 wt.% showing CNT pull-out, bridging, and CNTs embedded on GNP surfaces; (**c**–**e**) 0.5 wt.% and 0.5 wt.% illustrating CNT accumulation at grain boundaries, grain refinement, and boundary strengthening; (**f**) 0.5 wt.% and 0 wt.% depicting a large GNP rolled along an Al_2_O_3_ grain, enhancing interfacial contact and pull-out resistance [111].

Despite its benefits, thermal instability and poor dispersion at high processing temperatures make it difficult to effectively strengthen ceramics with CNTs and graphene [111]. According to Yazdani et al. [111], well-dispersed hybrid GNT (graphene nanoplatelets + carbon nanotubes) fillers allowed for near-full densification (>99%) of Al_2_O_3_ nanocomposites while also improving the microstructure, changing the mode of fracture from inter-granular to trans-granular, and greatly increasing flexural strength and fracture toughness. CNT and GNP dispersion in Al_2_O_3_–GNT nanocomposites following sintering is shown in Figure 4a–f. GNPs anchor around grains (Figure 4f), boosting pull-out energy, whereas CNT pull-out and bridging (Figure 4a,d) improve fracture toughness. CNTs embed on GNP flakes in Figure 4b, demonstrating a synergistic interaction. Grain borders exhibit CNT accumulation, which refines grains and strengthens interfaces, causing fracture to change from intra-granular to trans-granular (Figure 4c). While CNTs bridge grains because of their high aspect ratio and extended stretching before collapsing, GNPs mainly encourage grain refining and extensive inter-facial contact. When combined offers complimentary toughening mechanisms: bridging and boundary strengthening from CNTs, and anchoring and frictional resistance from GNPs. To avoid graphitization or oxidative degradation of carbon nanostructures during sintering, which can seriously impair their reinforcing efficiency, Balázsi et al. underlined the necessity of tailored processing methods [112]. Ceramic densification operations sometimes need high temperatures (>1200 °C), which must be carefully regulated or complemented by quick, low-temperature sintering methods [113,114].

Advanced densification methods like flash sintering, hot isostatic pressing, and spark plasma sintering (SPS) have been widely used to overcome these obstacles. Fast heating rates and brief rest times provided by SPS allow for high relative densities in the finished composite while maintaining the integrity of graphene and CNTs. These techniques improve the interfacial connection between the carbon fillers and the ceramic matrix while also reducing grain formation and porosity [115]. Emerging techniques utilizing hybrid or multiphase reinforcements, like pairing graphene with transition metal carbides or carbon nanotubes with SiC whiskers, were highlighted by Yin et al. [115]. By combining several reinforcing geometries and interfacial interactions, these hybrid systems provide synergistic toughening, which improves fracture resistance and thermal fatigue performance as compared to single-filler systems. Furthermore, it has been demonstrated that surface functionalization of carbon nanostructures with oxygen-containing groups or ceramic-compatible agents can improve dispersion uniformity and chemical compatibility inside ceramic matrices [116,117]. These ceramic nanocomposites’ increased tribological performance and mechanical resilience have expanded their range of applications to include biomedical implants, cutting tools, armor systems, and high-temperature insulators [118]. They are also being actively investigated for their potential in microelectronic packaging and additive manufacturing. The widespread use of graphene and CNT-reinforced ceramics will also be influenced by scalable processing, affordable carbon nanostructure production, and environmental stability in harsh environments [119]. The fracture-limited behavior of conventional ceramics has been successfully transformed into multifunctional systems with higher strength, durability, and operational versatility by incorporating carbon-based nanofillers into ceramic matrices.

### 3.4. Multifunctionality and Hybrid Nanostructures

The development of nanocomposites has expanded to include a wider concept of multifunctionality in recent years, going beyond traditional objectives like enhancing mechanical strength or thermal conductivity. The need for materials to simultaneously exhibit multiple performance attributes, such as mechanical robustness, thermal stability, electrical conductivity, barrier resistance, and biocompatibility, is driving this shift in advanced industries like aerospace, defense, biomedicine, and flexible electronics [120]. The hybridization of carbon nanotubes (CNTs) and graphene with other functional nanomaterials is a crucial tactic for attaining multifunctionality. New synergistic effects can be introduced by incorporating metal nanoparticles (like Cu, Ag), metal oxides (like ZnO, TiO_2_), and even organic or biodegradable polymers [121]. Hybrid carbon-based nanostructures embedded in functionally graded composites, for example, showed superior performance under harsh environments, including improved radiation shielding, thermal shock resistance, and impact energy dissipation—all crucial for aerospace and structural systems, according to Kumar et al. and Sarkar et al. [121,122]. It is challenging to create interconnected, multifunctional networks within the matrix using single-component reinforcements, but these hybrid fillers assist in doing so.

The advent of architecture nanocomposites, made possible by sophisticated fabrication methods like additive manufacturing (AM), direct ink writing (DIW), and 3D printing, is another revolutionary development [123,124,125]. These methods give exact control over the orientation and spatial arrangement of nanofillers, adding a new level of control over anisotropic characteristics. Wu et al. highlighted the application of 3D-printed graphene-CNT nanocomposites in energy harvesting, wearable electronics, and electromagnetic interference (EMI) shielding [125]. However, obtaining consistent and scalable multifunctional outputs during printing depends on managing the rheology of the ink, the dispersion of the nanofiller, and the alignment of the ink [126]. Simultaneously, a new era in nanocomposite design and prediction has been made possible by the merging of computational materials science and machine learning (ML) [127]. Barbaros et al. demonstrated how data-driven models can be used to predict the mechanical characteristics, interfacial stress buildup, percolation behavior, and dispersion states of nanofillers. The trial-and-error stage is greatly decreased by such approaches, which also make it possible to find the best combinations and architectures [127]. Researchers can now more accurately and efficiently create hybrid nanocomposites with customized functions for applications by utilizing large datasets and microstructural simulations [128]. Thus, a new generation of intelligent, multifunctional nanocomposite systems is being ushered in by the convergence of material hybridization, enhanced processing, and AI-guided optimization.

The performance of graphene- and carbon nanotube (CNT)-based nanocomposites is intrinsically governed by size-dependent electronic structure and the way nanoscale building blocks assemble into percolated networks within a host matrix. Unlike conventional reinforcements, nanocarbons exhibit electronic, mechanical, and thermal properties that are highly sensitive to lateral dimensions, thickness, aspect ratio, and defect density [129]. In graphene, reduction in lateral size and layer number induces quantum confinement effects, modifies the density of states near the Fermi level, and alters charge carrier mobility. Few-layer graphene and graphene nanoplatelets with reduced lateral dimensions often exhibit widened band gaps and increased edge-state contributions, which directly influence electrical conductivity, interfacial charge transfer, and sensing performance in nanocomposites [130]. Similarly, CNT diameter, chirality, and length determine whether the nanotube behaves as metallic or semiconducting, thereby controlling percolation behavior and electronic transport within composite systems [131].

**Figure 5:**
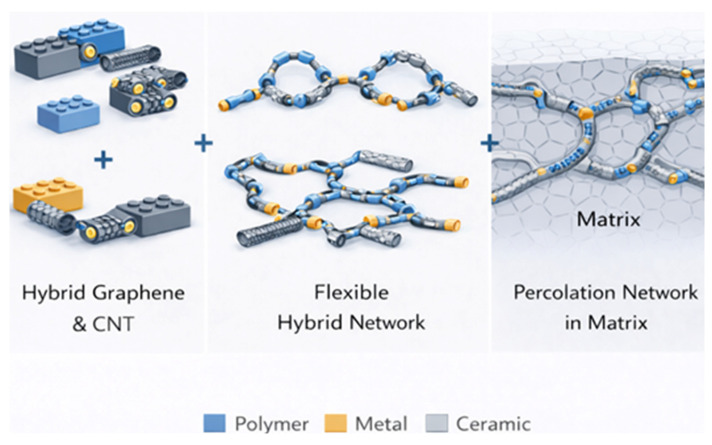
Schematic illustration of percolation and network formation in hybrid graphene/CNT nanocomposites, highlighting the building-block assembly of two-dimensional graphene platelets and one-dimensional carbon nanotubes into an interconnected conductive network within polymer, metal, and ceramic matrices, leading to reduced percolation thresholds and enhanced multifunctional performance.

From a structural standpoint, graphene sheets and CNTs can be regarded as complementary nanoscale building blocks, two-dimensional platelets and one-dimensional filaments that assemble into hierarchical networks. Graphene provides large-area conductive pathways and effective barrier properties, while CNTs act as bridging elements that connect adjacent graphene sheets, reducing inter-sheet contact resistance and lowering percolation thresholds (Figure 5) [132]. This building-block concept explains why hybrid graphene/CNT systems frequently outperform single-filler composites at equivalent filler loadings. Rather than acting independently, the two nanocarbon geometries cooperate to form multiscale networks capable of efficient load transfer, electron transport, and phonon conduction [133]. The spatial arrangement, overlap probability, and orientation of these building blocks strongly influence macroscopic properties, particularly electrical conductivity, EMI shielding effectiveness, and mechanical reinforcement.

Computational modeling has played a central role in elucidating how size and geometry govern network formation and functionality. Density functional theory (DFT) calculations have shown that graphene edge states, dopant incorporation, and curvature-induced strain in CNTs significantly affect electronic band structure and interfacial charge redistribution when embedded in polymer or metal matrices [134]. These electronic structure modifications influence tunneling barriers, contact resistance, and local electric fields at the filler–matrix interface. Molecular dynamics (MD) simulations further provide insight into how nanocarbon size and aspect ratio affect interfacial shear stress, load transfer efficiency, and debonding behavior under mechanical deformation [135]. Longer CNTs and larger graphene sheets generally promote more effective stress transfer but are also more prone to entanglement and agglomeration, highlighting a trade-off between reinforcement efficiency and processability.

Modeling-based approaches have also advanced the understanding of dispersion and percolation phenomena in nanocomposites. Percolation theory, combined with Monte Carlo simulations and coarse-grained MD models, has been used to predict critical filler concentrations required to establish conductive networks as a function of aspect ratio, orientation, and spatial distribution [136,137]. These studies consistently show that hybrid graphene/CNT systems achieve percolation at significantly lower filler loadings than single-filler systems due to enhanced connectivity and reduced excluded volume effects. Finite element modeling has further been applied to simulate stress distribution and interfacial load sharing within representative volume elements, revealing how stress concentrates at filler junctions and how network topology governs macroscopic mechanical response [138].

Interfacial stress development represents another critical area where modeling provides essential guidance. Multiscale simulations indicate that local stress amplification occurs at graphene edges, CNT ends, and filler junctions, particularly in stiff matrices such as ceramics or metals. Surface functionalization, filler alignment, and matrix compliance strongly influence stress relaxation mechanisms at these interfaces [139]. Phase-field and cohesive-zone models have been employed to predict crack initiation and propagation paths in graphene- and CNT-reinforced systems, demonstrating how nanocarbon networks deflect cracks, bridge microcracks, and dissipate energy through pull-out and sliding mechanisms [140]. These predictions correlate well with experimental observations of enhanced fracture toughness and fatigue resistance in hybrid nanocomposites.

Recent advances in data-driven and machine-learning-assisted modeling have further expanded predictive capabilities. By integrating descriptors such as filler size distribution, aspect ratio, surface chemistry, and processing parameters, machine learning models can predict dispersion quality, percolation thresholds, and effective mechanical or electrical properties with reduced experimental input [141]. Such approaches enable rapid screening of design spaces and facilitate optimization of graphene/CNT ratios for application-specific targets. Importantly, these models emphasize that optimal performance arises from balanced network architecture rather than maximum filler loading. Overall, modeling-guided insights into size-dependent electronic structure, building-block assembly, and network formation provide a unifying framework for rational nanocomposite design. By linking nanoscale geometry and electronic structure to macroscopic performance, these approaches reduce reliance on empirical trial-and-error and support the development of reproducible, scalable graphene/CNT nanocomposites tailored for structural, electronic, and multifunctional applications.

## 4. Fabrication Techniques and Process Integration

One of the biggest obstacles to the creation of high-performance multifunctional materials is the production of graphene and carbon nanotube (CNT)-reinforced nanocomposites. Graphene and carbon nanotubes (CNTs) tend to aggregate during composite processing because of their intrinsic anisotropy, strong van der Waals interactions, and huge surface-to-volume ratio. Strategies that guarantee uniform dispersion, maintain structural integrity, and foster robust interfacial interactions are necessary for incorporation into various matrices, whether they are ceramic, metallic, or polymeric [142]. As a result, numerous fabrication procedures have been created and improved upon, with notable variations based on the matrix and intended use. Current manufacturing processes for polymer matrix nanocomposites (PMNCs) are dominated by conventional techniques such melt mixing, solution blending, and in situ polymerization [143]. Because of its relative simplicity and accessibility of integrating functionalized nanofillers, solution blending is still a preferred method [144]. Before the polymer is dissolved and the solution is cast into films, graphene or CNTs are usually dispersed in organic solvents like N,N-dimethylformamide (DMF) or N-methyl-2-pyrrolidone (NMP), with the help of ultrasonication and surfactants [145]. This technique works well for polymers that dissolve easily in organic solvents and provide good initial dispersion. However, the research has moved toward greener and more commercially viable options due to worries about solvent toxicity, challenges with total solvent removal, and restricted scalability.

A solvent-free, scalable method that is more suited for industrial applications is melt mixing, which is frequently carried out via twin-screw extrusion [146]. This process involves compounding thermoplastic pellets and nanofillers at high temperatures while applying strong shear forces. Achieving uniform dispersion is challenging, particularly when working with entangled CNTs or aggregated graphene nanoplatelets, even though this method is ecologically friendly and provides continuous processing [147]. The surface functions added to encourage filler–matrix adhesion may also be weakened by the high processing temperatures. However, recent research has shown that precisely adjusting shear conditions can increase filler alignment and mechanical enhancement [148,149,150]. For instance, aligned carbon nanotubes (CNTs) in polyethylene matrices made by melt mixing have shown enhanced electrical percolation behavior and tensile characteristics, indicating the method’s potential when process parameters are adjusted [151]. Additionally, in situ polymerization has become popular, especially for high-performance engineering polymers and thermoset systems [152]. This process entails distributing nanofillers in monomer solutions and then polymerizing them chemically or thermally. During polymerization, functionalized fillers like carboxylate CNTs or amine-modified graphene oxide are frequently employed to start covalent bonding with the polymer chains, improving load transfer and interfacial adhesion [153]. When made using in situ procedures, epoxy, polyimide, and polyaniline matrices have demonstrated notable gains in mechanical strength, impact resistance, and thermal stability [154]. The final qualities, however, are extremely sensitive to the functional chemistry, dispersion quality, and degree of polymerization of the filler, and the procedure necessitates careful control over reaction conditions.

On the other hand, the low wettability of carbon-based nanofillers by molten metals and their possible reactivity at high temperatures present unique hurdles for metal matrix nanocomposites (MMNCs) [155]. For MMNC manufacture, powder metallurgy has become the most used method. In this case, metal powders are mixed with graphene or carbon nanotubes (CNTs), frequently by ball milling to enhance filler dispersion, then compaction and sintering [156]. By using this technique, the wettability problem is avoided, and high filler loadings can be incorporated. Agglomerates can be broken up and nanofillers can be embedded into the metallic matrix via high-energy ball milling. Nevertheless, excessive milling might weaken the fillers’ inherent qualities by harming the graphitic structure [157]. Temperature and environment control are essential throughout the sintering process to avoid oxidation or carbide formation, which can weaken the interface. Spark plasma sintering (SPS) has been used more often to improve densification and reduce filler degradation. SPS considerably lowers the thermal exposure of nanofillers by achieving fast sintering using pulsed DC current and uniaxial pressure [158]. This method reduces porosity in the final composite, encourages grain refinement, and maintains the structural integrity of graphene and CNTs. A thorough analysis revealed that because of their well-dispersed reinforcements and refined microstructures, aluminum-graphene and copper-CNT composites made by SPS have higher hardness, tensile strength, and thermal conductivity [159]. The primary disadvantages of SPS are its high equipment cost and restricted scalability for big parts; however, these issues are being steadily resolved by developments in die design and multi-sample processing.

**Figure 6:**
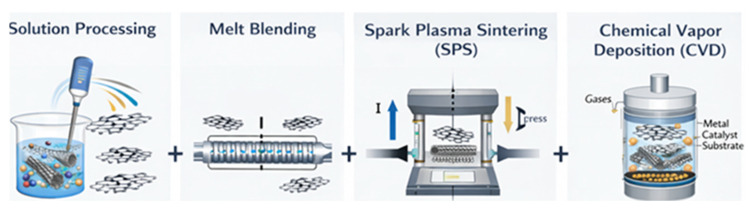
Schematic illustration of representative fabrication and processing routes for graphene/CNT nanocomposites, including solution processing, melt blending, spark plasma sintering (SPS), and chemical vapor deposition (CVD)-assisted approaches, highlighting their role in achieving uniform dispersion, interfacial bonding, and scalable composite production across different matrix systems.

Chemical vapor deposition (CVD) has emerged as one of the most influential synthesis routes for graphene and carbon nanotubes (CNTs), particularly in the context of achieving controlled morphology, high purity, and tunable structural properties. However, the widespread adoption of CVD-derived nanocarbons in nanocomposite manufacturing has historically been constrained by high capital costs, batch processing limitations, and restricted throughput (Figure 6) [160]. Recent developments in atmospheric-pressure CVD (APCVD), plasma-enhanced CVD (PECVD), and roll-to-roll (R2R) processing have significantly improved the economic and industrial feasibility of CVD-grown graphene/CNT systems [161]. Atmospheric-pressure CVD (APCVD) represents a major step toward cost reduction by eliminating vacuum systems and simplifying reactor design. APCVD enables continuous growth of graphene and CNTs on large-area metallic substrates such as copper and nickel foils, making it particularly attractive for nanocomposite applications where large-volume production and moderate defect tolerance are acceptable [162]. While APCVD-grown graphene may exhibit higher defect densities or multilayer formation compared to low-pressure CVD, such features can be advantageous in composite systems by enhancing interfacial bonding, mechanical interlocking, and load transfer within polymer and metal matrices.

Plasma-enhanced CVD further expands processing flexibility by enabling nanocarbon growth at substantially reduced temperatures through plasma-activated precursor decomposition. This low-temperature capability is especially relevant for nanocomposite fabrication involving temperature-sensitive substrates, including polymers, metal powders, and coated reinforcements. PECVD-grown CNTs often exhibit vertically aligned or highly entangled morphologies, which can be directly integrated into composite architectures to improve percolation behavior, electrical conductivity, and interfacial shear strength [163]. Moreover, plasma-assisted processes allow partial control over defect density and functional group incorporation, which can be exploited to tailor matrix–filler interactions without extensive post-synthesis functionalization. Roll-to-roll CVD has emerged as a transformative approach for industrial-scale graphene and hybrid graphene/CNT production. By enabling continuous synthesis on flexible metallic foils, R2R processing significantly enhances throughput while lowering per-unit production cost. Sequential or simultaneous growth of graphene and CNTs within roll-to-roll systems has enabled the fabrication of hierarchical nanocarbon architectures that combine the high surface area and barrier properties of graphene with the bridging and network-forming capabilities of CNTs [164]. Such hybrid architectures are particularly advantageous for nanocomposites requiring multifunctional performance, including electrical conductivity, EMI shielding, and thermal management. Despite these advances, important trade-offs remain between material quality, production rate, and cost. High-throughput APCVD and R2R processes may yield increased defect densities, catalyst residues, or non-uniform nanocarbon structures, which can limit electronic performance but are often acceptable or even beneficial in structural and multifunctional nanocomposites [165]. Consequently, the suitability of CVD-derived graphene and CNTs must be evaluated in an application-specific context rather than solely based on idealized material properties. Overall, the evolution of APCVD, PECVD, and roll-to-roll CVD technologies reflects a clear transition from laboratory-scale synthesis toward industrially viable nanocarbon production, reinforcing the practical relevance of CVD-grown graphene/CNT systems for next-generation nanocomposite manufacturing.

Friction stir processing (FSP), a solid-state method in which a rotating tool stirs the surface of a metal substrate containing nanofillers, is a novel alternative in MMNC processing [166]. By promoting homogeneous dispersion and mechanical bonding without melting the matrix, the extreme plastic deformation prevents the fillers from degrading thermally. FSP works especially well for surface modification, allowing for the formation of functionally graded layers with improved resistance to corrosion and wear. Although the process is still limited to specific areas and is not yet practical for large-scale production, aluminum-CNT made via FSP has shown notable gains in fatigue life and thermal stability [167]. Because of their brittleness and high sintering temperatures, ceramic matrix nanocomposites (CMNCs) need to be processed with extra care. Conventional techniques like hot pressing and hot isostatic pressing (HIP) provide controlled densification under pressure and heat, producing dense microstructures with better mechanical qualities. These techniques work especially well with matrices made from silicon carbide (SiC), alumina (AlO_3_), and zirconia (ZrO_2_) [168]. However, the high processing temperatures frequently cause reactions that result in undesirable phases like silicon carbide or aluminum carbide, or they damage carbon-based reinforcements. To get around this, protective coatings and surface functionalization are applied to the nanofillers to increase wettability and preserve stability [169].

For ceramic-based composites, SPS has also demonstrated great potential. By consolidating materials at lower temperatures and in shorter amounts of time, it considerably slows grain formation and helps maintain the reinforcements’ nanostructure. In addition to improved fracture toughness brought about by crack deflection and bridging, AlO_3_-graphene nanocomposites made via SPS have demonstrated functional qualities including electrical conductivity, creating new opportunities for structural electronics and sensing applications [170]. Despite its advantages, SPS is still expensive and only used in lab settings, while recent advancements in hybrid SPS-assisted 3D formation and scale-up show promise. Additive manufacturing (AM) techniques have emerged as innovative platforms for nanocomposite fabrication with the growth of advanced manufacturing. The most researched methods for polymer-based systems are selective laser sintering (SLS), fused filament fabrication (FFF), and direct ink writing (DIW) [171]. Nanocomposite inks may be extruded thanks to DIW, which gives exact control over filler alignment and architecture. The anisotropic qualities of CNTs and graphene are improved by shear-induced orientation during printing, which is advantageous in applications that call for mechanical reinforcement or directional conductivity [172]. DIW’s effectiveness, however, depends on the ink’s rheological characteristics and the stability of the nanofillers inside the matrix. Conversely, FFF and SLS use pre-made filaments or powders that have been impregnated with nanofillers. By incorporating electrical, thermal, or sensor capabilities directly into structural components, these techniques allow for the creation of complicated geometries [173]. In 3D-printed items, graphene-infused PLA or carbon nanotube-infused nylon filaments have shown enhanced mechanical strength, thermal conductivity, and EMI shielding efficacy [174]. However, post-processing procedures like annealing or infiltration are frequently needed to improve final characteristics, and interfacial bonding and filler alignment continue to be significant obstacles.

Functionally graded and architectural materials, in which the distribution, orientation, or kind of nanofiller vary spatially within the composite, are another emerging fabrication technique. This design makes it possible to create multipurpose parts with customized electrical conductivity, stress distribution, or thermal expansion. Composites having varying graphene content between layers, for example, can serve as materials for thermal interfaces with regulated heat flux pathways. Usually, layer-by-layer deposition procedures or sequential casting and curing methods are used to achieve such gradients [175]. Because of their complexity, these structures require sophisticated simulation tools and in-process monitoring to guarantee performance and reproducibility. Combining various manufacturing approaches to get around individual limits is becoming more important from the standpoint of process integration. A compromise between scalability, performance, and affordability is made possible by hybrid techniques such melt mixing followed by compression molding or powder metallurgy mixed with surface FSP [176]. To guarantee constant quality in large-scale production, developments in automation, robotic mixing, and machine learning-guided process optimization are also being investigated. Additionally, the creation of pre-functionalized nano-filler masterbatches provides a workable technique to integrate carbon nanostructure into conventional processing lines without requiring major equipment adjustments.

## 5. Mechanisms and Interfacial Interactions

**Figure 7:**
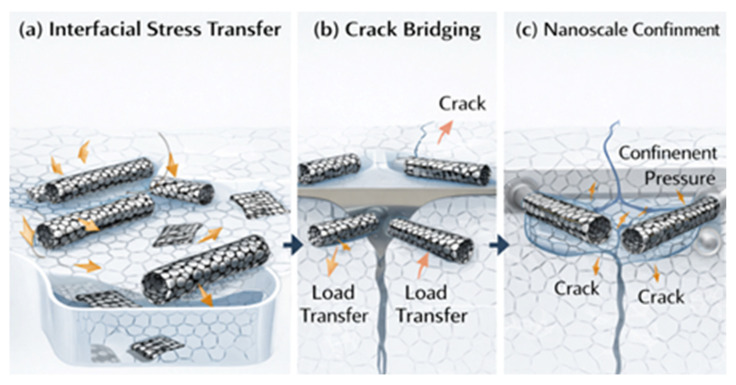
Schematic illustration of interfacial load-transfer and strengthening mechanisms in hybrid graphene/CNT nanocomposites, highlighting (**a**) efficient stress transfer across the filler–matrix interface, (**b**) crack bridging and deflection by CNTs and graphene platelets, and (**c**) nanoscale confinement effects that suppress crack propagation and enhance mechanical reinforcement.

The methods of stress transfer, load distribution, and the type of interfacial contact between the nanofillers and the surrounding matrix essentially control the improved multifunctional performance of nanocomposites reinforced with graphene and carbon nanotubes (Figure 7a–c). The degree to which the exceptional intrinsic qualities of carbon nanostructures can be converted into macroscale performance in nanocomposites is largely determined by the contact [177]. Stress continuity, effective load transfer, and the avoidance of filler slippage or pull-out during mechanical deformation are all made possible by effective interfacial bonding. Furthermore, the interface has a major impact on the degradation mechanisms under environmental stimuli, crack propagation behavior, and electrical and thermal conductivity routes [178]. The strengthening and functional enhancement observed in graphene- and CNT-reinforced nanocomposites are strongly governed by size-dependent mechanisms operating at the nanoscale [179]. Grain refinement induced by uniformly dispersed nanocarbon fillers increases dislocation density and restricts dislocation motion, thereby enhancing strength through combined Hall–Petch and Orowan-type mechanisms. In hybrid systems, graphene platelets act as effective grain boundary pinning agents, while CNTs bridge adjacent grains and promote load transfer across interfaces. These synergistic effects are particularly evident in spark plasma sintering (SPS)-processed systems, where controlled densification suppresses porosity and enables efficient stress redistribution across the filler–matrix interface [180]. Beyond mechanical reinforcement, these nanoscale size effects also influence functional properties such as electrical and thermal transport by modifying percolation pathways and interfacial resistance. The interaction of nanocarbon fillers with grain boundaries and phase interfaces creates interconnected networks that enhance conductivity while maintaining structural integrity [181]. Consequently, the combined influence of grain refinement, interfacial architecture, and processing-induced densification underscores the importance of nanoscale design strategies in achieving high-performance graphene/CNT nanocomposites.

To fully utilize these nanocomposite systems, a mechanistic understanding of the interaction modes and customization of the interfacial architecture are essential. Interfacial shear stress generated at the nanofiller–matrix interface is the main load transmission mechanism in polymer matrix nanocomposites [182]. Because of their atomically clean surfaces and chemical inertness, pristine graphene and carbon nanotubes have poor interfacial adhesion with most polymers [183]. This results in poor stress transfer and underutilization of the high modulus of the nanofillers due to slippage and debonding under applied stresses. Functionalization techniques are frequently used to overcome such drawbacks. Covalent functionalization allows for chemical bonding with the polymer chains by introducing reactive groups like -COOH, -OH, or -NH_2_ onto the nanofiller surface. Although this improves load transmission, the electrical and thermal characteristics deteriorate because of the covalent changes, which partially disrupt the sp^2^ hybridized conjugated π-system [184]. Conversely, non-covalent functionalization with polymers, surfactants, or π–π stacking interactions maintains the graphitic structure while increasing interfacial compatibility and dispersion [185]. To improve wetting and entanglement with the polymer matrix, poly-vinyl pyrrolidone (PVP), polystyrene sulfonate (PSS), and pyrene derivatives have been extensively investigated for their capacity to absorb onto nanofillers via van der Waals or π interactions [186].

Among the main toughening processes seen in graphene- and CNT-reinforced polymers are interfacial debonding, pull-out, and crack-bridging. Nanofillers efficiently transfer the load from the matrix to the filler under tensile stress, whereas those with weak interfaces often debond and pull out, wasting energy and postponing catastrophic failure [187]. During crack propagation, graphene sheets wrinkle and fold, blunting the crack tip and promoting extrinsic toughening [188]. According to their aspect ratio and interfacial bonding, CNTs also show pull-out, bridging, and even breaking [189]. The role of interfacial stress concentration and load transfer in graphene/CNT nanocomposites is further clarified by modeling-based analyses that explicitly link filler size, aspect ratio, and network topology to local stress amplification and relaxation mechanisms [190]. Multiscale simulations demonstrate how graphene edges, CNT junctions, and hybrid filler intersections govern interfacial shear stress and crack deflection behavior. According to the critical length theory, which emphasizes the significance of both aspect ratio and dispersion, the length of CNTs should surpass a specific threshold for efficient stress transfer [191]. The idea that interfacial shear stress and van der Waals energy dissipation at the interface are essential for improving the energy absorption capacity of nanocomposites under mechanical loading is further supported by molecular dynamics simulations [192]. Because of metallurgical phenomena including interfacial reactions, carbide formation, and diffusion bonding, the nature of interfacial interaction in metal matrix nanocomposites is more complicated [193]. Because of their low surface energy and lack of chemical affinity, graphene and carbon nanotubes are typically poorly wettable by molten metals like aluminum, copper, or magnesium. Poor adhesion and the existence of cavities at the contact result from this. Reactions between the metal and carbon nanostructures can produce brittle and hygroscopic metal carbides like Al3C3 at high processing temperatures, particularly during casting or sintering [194]. These stages jeopardize the composites’ ability to withstand corrosion and maintain their mechanical integrity. As demonstrated in Ti–C systems, where TiC production at the interface enhances bonding and thermal stability, a moderate amount of interfacial reactivity can occasionally be advantageous by attaching the nanofillers to the matrix [195].

Dopants and residual impurities play a decisive role in regulating the multifunctional performance of graphene- and carbon nanotube (CNT)-reinforced nanocomposites, yet their influence is frequently underrepresented in experimental discussions [196]. Intentional heteroatom doping commonly involving nitrogen, boron, sulfur, and phosphorus has emerged as an effective route for tailoring the electrical, magnetic, and electromagnetic interference (EMI) shielding properties of nanocarbon fillers without fundamentally altering their morphology [197]. Nitrogen-doped graphene and CNTs introduce electron-rich active sites that enhance carrier concentration, electrical conductivity, and interfacial charge transfer, making them particularly suitable for conductive polymer composites, sensors, and energy-storage devices [198]. In contrast, boron doping induces p-type behavior by creating electron-deficient regions, enabling controlled semiconducting characteristics and improved compatibility with ceramic and metal matrices where charge redistribution at the interface governs performance [199]. Magnetic dopants such as iron, cobalt, and nickel introduce additional magnetic loss mechanisms that significantly enhance EMI shielding effectiveness when combined with conductive nanocarbon networks. In hybrid polymer nanocomposites, these dopants promote synergistic attenuation through simultaneous dielectric loss, magnetic hysteresis, and eddy current dissipation. Such effects are especially pronounced in doped graphene systems, where the two-dimensional structure facilitates extended conductive pathways while embedded magnetic species broaden the frequency range of effective shielding [197,198,199]. However, excessive dopant concentrations may induce structural disorder, disrupt the sp^2^ carbon lattice, and reduce carrier mobility, emphasizing the importance of controlled dopant incorporation.

**Figure 8:**
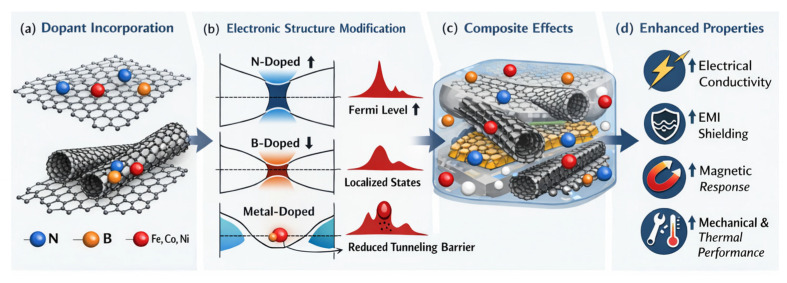
Schematic of dopant-engineered hybrid graphene–CNT composites. (**a**) Incorporation of N, B, and metal dopants (Fe, Co, Ni) into the carbon framework; white spheres denote carbon atoms and black spheres represent metal dopants. (**b**) Doping-induced electronic structure modification with Fermi-level shifts and reduced tunneling barriers. (**c**,**d**) Formation of conductive networks leading to enhanced electrical, EMI shielding, magnetic, mechanical, and thermal properties.

In addition to intentional doping, unintentional impurities—particularly residual catalyst particles and oxygen-containing functional groups—strongly influence nanocomposite behavior. Catalyst residues originating from chemical vapor deposition or arc-discharge synthesis can act as local stress concentrators in mechanical loading and as galvanic corrosion sites in metal matrix systems [200]. Conversely, in polymer nanocomposites, such metallic residues may contribute beneficially to magnetic response and EMI shielding. Oxygen functional groups introduced during oxidative exfoliation or chemical functionalization improve nanocarbon dispersibility and interfacial adhesion with polar matrices by enabling hydrogen bonding or covalent interactions [201]. However, these groups disrupt the conjugated π-electron network, leading to reduced electrical and thermal conductivity. Achieving an optimal balance between functionalization-driven processability and retention of intrinsic nanocarbon properties remains a central challenge in composite design. Modeling-guided approaches have become increasingly important for predicting and rationalizing the effects of dopants and impurities on the electronic structure and interfacial behavior of graphene and CNTs [202]. Density functional theory (DFT) calculations have shown that substitutional dopants alter the local density of states, band structure, and Fermi level position, directly influencing carrier mobility and interfacial charge transfer with surrounding matrices. Molecular dynamics simulations further reveal how dopant-induced defects modify interfacial shear stress, load transfer efficiency, and phonon scattering, thereby affecting mechanical reinforcement and thermal transport (Figure 8) [203]. These computational insights provide a rational framework for selecting dopant species and concentrations tailored to specific application requirements, such as maximizing EMI shielding, enhancing piezoresistive sensitivity, or improving thermal management.

Processing conditions critically mediate dopant effectiveness and impurity stability. High-temperature processing routes, such as conventional sintering or casting, may induce dopant diffusion, clustering, or carbide formation, particularly in metal and ceramic matrix systems, thereby altering both microstructure and functional performance [204]. In contrast, low-temperature routes such as solution processing, plasma-assisted deposition, and spark plasma sintering better preserve dopant configurations and interfacial chemistry. Emerging machine-learning-assisted frameworks that integrate synthesis parameters, dopant chemistry, and processing conditions are now enabling predictive correlations between processing, dopant distribution, and composite properties. Such data-driven approaches offer a promising pathway toward reproducible, application-specific optimization of graphene/CNT nanocomposites.

Surface engineering approaches are used to reduce undesirable reactions. It has been demonstrated that coating nanofillers with a protective ceramic or metallic shell, such as SiO_2_, AlO_3_, or Ni, can improve interfacial bonding and stop deterioration [205]. By improving wettability with the metallic matrix and acting as diffusion barriers, these coatings create stronger surfaces. Furthermore, adding alloying metals like magnesium or titanium can improve the composite’s microstructure and increase wettability [206]. Another important process via which the physical trapping of graphene or carbon nanotubes at grain boundaries or dislocation locations aids in grain refinement and the obstruction of dislocation motion is the mechanical anchoring effect of nanofillers, sometimes referred to as the Orowan strengthening mechanism [207]. The yield strength, hardness, and wear resistance of MMNCs are enhanced by the synergistic effects of load transfer, thermal mismatch strain, and grain boundary pinning. Because of the high sintering temperatures and brittleness of the matrix, ceramic matrix nanocomposites provide special interfacial problems [208]. When exposed to high temperatures in oxidizing or reactive atmospheres, graphene and carbon nanotubes are susceptible to deterioration or graphitization [209]. It is therefore challenging to maintain the integrity of the carbon nanostructures while guaranteeing robust interfacial adhesion. However, because powder-processed ceramics are inherently rough and porous, mechanical interlocking frequently improves ceramic–carbon surfaces. Without chemical bonding, this mechanical interlocking in conjunction with van der Waals interactions can produce mild adhesion [210]. Additionally, by adding reactive moieties that can create strong covalent or ionic interactions during sintering, surface functionalization of graphene oxide (GO) with silane coupling agents or metal alkoxides improves bonding with ceramic matrices [211].

Graphene and CNTs serve as bridging agents, branch inducers, and crack deflectors, according to a fracture mechanics investigation of CMNCs. Graphene sheets can delaminate and slide under stress, wasting energy and altering crack paths because of its high aspect ratio and flexibility. Conversely, CNTs can effectively increase fracture toughness by bridging microcracks and maintaining load-bearing capacity across expanding cracks [212]. The filler–matrix interface has a significant impact on the magnitude of these mechanisms. While excessively strong bonding may result in brittle failure via filler fracture, weak surfaces may permit premature pull-out and void formation. Therefore, to maximize toughness without sacrificing stiffness and strength, the ideal balance between adhesion and mobility must be achieved. Another important aspect in determining the effective thermal conductivity of nanocomposites is interfacial thermal resistance, also known as Kapitza resistance [213]. The mismatch in phonon spectra between the nanofillers and the matrix causes phonon scattering at the interface, which lowers overall heat transport even though graphene and carbon nanotubes have excellent intrinsic conductivity. Phonon coupling across the contact can be modulated by functionalization and interface engineering; for example, covalently bonded interfaces offer superior phonon transport channels but may decrease electrical conductivity because of interrupted conjugation [214]. Alternatively, a balanced pathway for thermally conductive yet electrically insulating nanocomposites—desirable for electronic packaging and thermal management—is provided by bridging molecules or interface modifiers that improve phonon coupling without interfering with electronic delocalization [215].

Interfacial interactions are the source of electrically conductive networks in nanocomposites as well. Close contact or tunneling lengths between neighboring fillers are necessary for the creation of percolative routes [216]. Low-resistance network development is facilitated by surface changes that increase dispersion and decrease aggregation [217]. Dielectric behavior is also influenced by interfacial polarization at the matrix–filler interface, particularly in polymer nanocomposites. Functional groups or ionic moieties increase interfacial polarization, which improves energy storage and dielectric characteristics and makes these composites appropriate for capacitive and sensing applications [218]. Interphase engineering, hierarchical filler structures, and hybrid interfacial methods that mix covalent and non-covalent interactions have drawn attention in recent years. A novel paradigm for interface optimization is provided by the idea of a designed interphase, which consists of a gradient in the composition, stiffness, or polarity between the matrix and nanofiller [219]. These interphases can improve load transfer, mediate stress concentration, and encourage multifunctionality. Emerging strategies to customize interfacial chemistry and mechanics for next-generation nanocomposites include core–shell fillers, 3D hierarchical carbon networks, and bio-inspired adhesion motifs [220]. As a result, the interface controls the mechanical, thermal, electrical, and chemical performance limits of graphene and CNT-reinforced nanocomposites. The effectiveness of stress transfer, crack resistance, conductivity, and durability are determined by the design of interfacial interactions through functionalization, coating, alloying, or interphase engineering [221]. A thorough understanding of these principles opens the door for new applications in structural, electrical, and energy systems where multifunctional performance is crucial, in addition to assisting in the logical design of superior nanocomposites.

## 6. Comparative Performance Analysis and Structure–Property Relationships

### 6.1. Mechanical Performance Enhancement Across Matrix Systems

Mechanical performance improvements reported across polymer, metal, and ceramic nanocomposites are closely linked to nanoscale dispersion and size effects rather than filler content alone. Studies summarized in Table 1 demonstrate that relatively low nanocarbon loadings can induce substantial gains in strength and hardness when grain refinement, interfacial bonding, and percolation networks are optimized. Conversely, excessive filler addition often leads to agglomeration and interfacial defects, offsetting potential gains. These findings emphasize that processing–structure–property correlations, rather than absolute filler concentration, govern the mechanical response of graphene/CNT-based nanocomposites [222]. A key component of nanocomposite research is the mechanical reinforcement of host matrices with the addition of graphene and carbon nanotubes (CNTs). These nanomaterials are perfect reinforcements for polymer, metal, and ceramic matrices because of their remarkable tensile strength, stiffness, and high aspect ratios. Numerous investigations have repeatedly shown that, as long as the nanofillers are well distributed and bound at the interface, even tiny weight fractions (usually less than 1 weight percent) of graphene and carbon nanotubes (CNTs) can result in notable increases in mechanical properties [223,224,225]. The improvement in tensile strength and modulus is most noticeable in polymer matrices, especially epoxy, polycarbonate (PC), and polypropylene (PP). For example, adding well-dispersed graphene nanoplatelets (GNPs) to epoxy matrices has improved tensile strength by more than 150% when compared to base polymer [226]. In a similar vein, CNTs enhance strength, ductility, and elongation at break in PC and PP matrices. With the CNTs serving as load-bearing structures during deformation, these improvements result from effective stress transfer between the matrix and the nanofillers [227]. By creating chemical linkages or enhancing compatibility between the nanofillers and matrix, functionalization procedures like carboxylation and amination further improve interfacial adhesion [228]. Multiple strengthening mechanisms are introduced by the incorporation of graphene or carbon nanotubes (CNTs) in metal matrix nanocomposites (MMNCs), particularly those based on lightweight metals like magnesium (Mg) and aluminum (Al). These consist of dislocation hindrance, grain boundary pinning, load transfer, and Orowan looping. With as little as 0.5 weight percent CNTs, experimental research has demonstrated yield strength enhancements of 30–50%, especially when sophisticated consolidation techniques like mechanical alloying and spark plasma sintering (SPS) are used [229]. Nanofillers aid in the refinement of the grain structure and the limitation of dislocation movement, both of which contribute to the strengthening behavior of the Hall-Petch type [230]. Additionally, fractographic investigations verify that the nanofillers are successful in preventing the spread of cracks, which increases the composite’s toughness and strength [231]. Graphene and carbon nanotubes (CNTs) significantly improve fracture toughness in ceramic matrix nanocomposites (CMNCs), which are often brittle by nature. This is particularly noteworthy in zirconia (ZrO_2_) and alumina (AlO_3_) systems, where gains in fracture toughness of up to 60% have been reported [232]. Crack bridging, CNT pull-out, interfacial debonding, and frictional sliding are toughening mechanisms in these systems that release energy when cracks spread [233]. These mechanisms were confirmed by scanning electron microscopy (SEM), which showed how nanofillers align and interact at the fracture tip to prevent crack opening and support structural integrity under stress [234]. When taken as a whole, these results highlight how important graphene and carbon nanotubes are for mechanical enhancement in a variety of matrices. Filler dispersion, interfacial bonding, and the synergy between the reinforcement and the matrix structure are the main factors that determine increased mechanical performance in polymers, metals, and ceramics.

Although graphene and carbon nanotubes (CNTs) have been incorporated into nanocomposite systems to enable remarkable performance advancements across a variety of materials, a nuanced understanding of the inherent performance trade-offs is necessary for the practical translation of these materials into industrial applications. Complex interdependencies between filler loading, processing limitations, interfacial interactions, and final property requirements frequently lead to these trade-offs. The increase in viscosity and processing difficulty at large nanofiller loadings is one of the most common limitations in polymer nanocomposites [243]. Defects, anisotropy, or brittleness in the finished composite might result from problems such nanofiller agglomeration, poor wetting, and inadequate dispersion that occur when the volume percentage of graphene or CNTs rises above a threshold (usually ~2–5 wt.%) [244]. If strong interfacial bonding is not achieved, an excessive amount of carbon nano-filler in metal matrix nanocomposites (MMNCs) might impair ductility and serve as crack initiation sites. An ideal filler content typically between 0.5 and 2.0 weight percent—is required to achieve a balance between tensile strength, fracture toughness, and manufacturability, according to Moghadam et al. and Gurnani et al. [245,246].

In high-performance industries, it is critical to adapt nanocomposite qualities according to specific applications. Materials that provide lightweight construction, improved fatigue resistance, impact tolerance, and vibrational dampening, as well as resistance to temperature cycling, UV radiation, and oxidative degradation, are necessary for aerospace and automotive applications [247]. Aerospace-grade CNT-reinforced polymer composites were examined by Iqbal et al., who showed how they could reduce microcracking and preserve structural integrity under varying mechanical and thermal stresses [248]. However, biomedical applications and consumer electronics place a higher priority on features like flexibility, environmental safety, recyclability, and biocompatibility. According to Cheng et al., functionalized nanostructures and biocompatible matrices are required to adapt graphene-based systems for stretchable electronics and drug delivery platforms [249]. Green processing technologies, solvent-free dispersion processes, and recyclable or biodegradable matrices are becoming more popular as sustainability becomes a critical factor. To lessen the environmental impact and promote circular material economies, researchers have emphasized how urgent it is to incorporate eco-friendly synthesis approaches [250]. For wide-scale adoption, it is essential that future research continues to balance high performance with process efficiency, cost-effectiveness, and environmental responsibility, tailored to the specific performance criteria of target industries. Table 1 summarizes the key literature on graphene and CNT-reinforced nanocomposites prepared by different routes.

### 6.2. Thermal Conductivity and Stability Enhancement

Advanced materials must have thermal conductivity, especially for high-performance uses in the automotive, electronics, aerospace, and energy industries. The necessity for materials with improved thermal conductivity grows as gadgets get smaller and more potent to guarantee effective heat dissipation and system dependability. Because of their remarkable intrinsic thermal conductivities, nanocarbon fillers like graphene and carbon nanotubes (CNTs) have become extremely effective thermal reinforcements in this context [251]. Conventional thermally conductive materials are greatly outperformed by graphene, which has a thermal conductivity of around 5000 W/mK, and carbon nanotubes (CNTs), which have a thermal conductivity of 3000–3500 W/mK. These nanomaterials significantly increase the composite’s total thermal conductivity when added to polymer matrices [252]. The formation of ongoing, percolated heat networks inside the insulating matrix is crucial. Lian et al. [252] showed that epoxy composites, which naturally have low thermal conductivity (~0.2 W/m·K), may obtain values as high as 2.1 W/m·K with just 5 wt.% graphene loading. The development of highly interconnected graphene routes that promote phonon transport across the matrix is responsible for this increase. Thermal performance is further optimized using hybrid nanofiller systems that include graphene (2D) and carbon nanotubes (1D) [253]. These systems make use of the structural synergy between the fillers—CNTs reduce thermal interface resistance and promote more effective heat transfer by bridging the gaps between graphene layers [254]. Thermal boundary resistance (TBR), which is frequently a limiting issue in composite systems, is significantly reduced because of the enhanced phonon coupling across interfaces. Controlled filler orientation is used to obtain further improvements in thermoplastic matrices such as polyimide and polyethylene terephthalate (PET) [255]. Anisotropic thermal conduction can be produced by aligning graphene sheets along the direction of heat flow using manufacturing processes like 3D printing, melt extrusion, or uniaxial drawing [256]. Applications where targeted and directed heat evacuation are crucial, such as flexible electronics and thermal interface materials, benefit greatly from this kind of directionality.

Metal matrix composites also demonstrate the advantages of graphene and carbon nanotubes. As little as 1.5 vol% graphene can increase the thermal conductivity of copper (Cu)-based systems, which are commonly used in electronic heat sinks and packaging, to over 550 W/m·K without sacrificing electrical conductivity or mechanical robustness [257]. Improved phonon transport at the metal–nanocarbon interface and reduced TBR because of the close contact and advantageous acoustic impedance matching are the main mechanisms. The use of graphene and carbon nanotubes (CNTs) improves thermal conductivity and thermal shock resistance for ceramic matrix composites, such as those based on silicon carbide (SiC) or alumina (Al_2_O_3_) [258]. In high-temperature environments, where rapid thermal cycling can cause cracking, this is crucial. Nanocarbon fillers increase the durability of the composite by bridging microcracks and dispersing heat more evenly. The potential application of 3D-printed graphene-ceramic hybrid materials in wearable electronics and thermal shielding is reported by Ali et al. [259], suggesting a trend toward multifunctional design in thermal management applications. As a result, graphene and carbon nanotubes greatly improve the thermal performance of metals, polymers, and ceramics, making them essential components of contemporary thermal management systems. They are perfect candidates for next-generation high-performance materials because of their adjustable qualities, compatibility with sophisticated manufacturing processes, and synergy with different matrices.

### 6.3. Electrical Properties and Functional Tailoring

**Figure 9:**
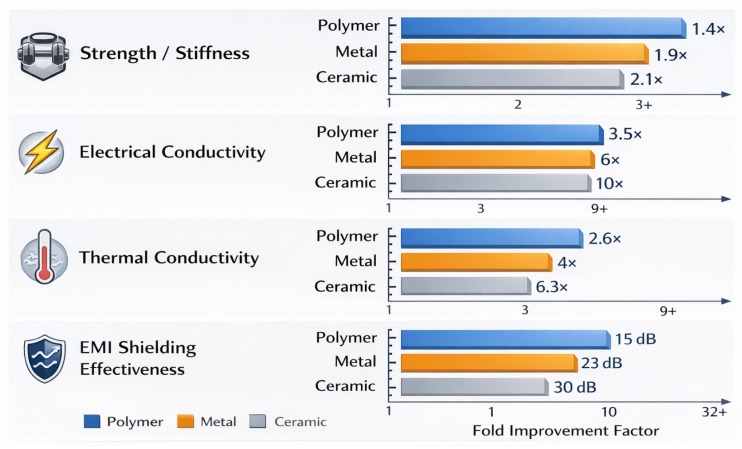
Comparative schematic chart summarizing relative enhancements in mechanical, electrical, thermal, and electromagnetic interference (EMI) shielding properties of graphene/CNT nanocomposites across polymer, metal, and ceramic matrices. The values shown represent normalized, literature-consistent trends intended to highlight comparative performance improvements rather than exact quantitative data for a specific material system.

Nanomaterials based on carbon, such as graphene and carbon nanotubes (CNTs), have exceptional inherent electrical conductivities, usually between 10^4^ and 10^5^ S/cm, and remarkable carrier mobilities (up to 200,000 cm^2^/V·s for suspended graphene) [260]. Since they possess these qualities, they are perfect for improving the electrical performance of matrices that would otherwise be insulating, like ceramics, elastomers, and thermosetting polymers. At very low filler loadings (~0.1–0.5 wt.%), the introduction of a percolating network of CNTs or graphene sheets in polymeric systems promotes the beginning of electrical conductivity [261]. This percolation threshold is dependent on the nanofillers’ surface functionalization, dispersion quality, and aspect ratio. For example, Kausar et al. found that adding merely 0.25 weight percent graphene to polymethyl methacrylate (PMMA) increased electrical conductivity by six orders of magnitude, from about 10^−13^ to about 10^−2^ S/cm, changing the polymer from an insulator to a semiconductor [262]. This adjustable conductivity has been used in several functional areas, including touch-sensitive devices, antistatic coatings, and electromagnetic interference (EMI) shielding. CNTs are frequently used in silicone and polyurethane (PU) matrices to produce piezoresistive composites, especially multi-walled varieties. These materials are ideal for biomedical patches, wearable strain sensors, and soft robotics because they show resistance changes in response to applied pressure or strain [263]. Their mechanical durability, great flexibility, and steady conductivity under dynamic loading cycles make them suitable for incorporation into smart fabrics and deformable electronics. At low strain regimes, the strain-sensing sensitivity (gauge factor) in these systems can reach values more than 100, with millisecond response and recovery periods. In metal matrix nanocomposites (MMNCs), graphene or carbon nanotubes (CNTs) play a more complex role, even if base metals like copper, aluminum, and magnesium already have excellent conductivity. Because of their inclusion, electrical resistance can be adjusted to fit uses, including energy harvesting layers, thermoelectric modules, or resistive heating elements [264]. For instance, ballistic electron transport along the CNTs gives Cu–CNT composites a better capacity to carry current, with additional advantages in thermal control [265]. By combining corrosion resistance, electrical conductivity (~10^6^ S/m), and even catalytic capabilities that are helpful in hydrogen evolution electrodes and microelectronic interconnects, hybrid MMNCs, including Cu–CNT–Ag systems, have shown multifunctional performance [266]. Because of their ionic-covalent bonding nature, ceramic matrices are naturally insulating; nevertheless, the addition of conductive nanofillers greatly improves them. When graphene or carbon nanotubes (CNTs) are added to silicon carbide (SiC), zirconia (ZrO_2_), or alumina (AlO_3_), the composite resistivity can be reduced by many orders of magnitude, making them suitable for use in high-temperature sensors and electro ceramics [267]. For example, graphene–ZrO_2_ composites maintained mechanical strength and thermal stability despite demonstrating resistivities as low as ~10^−3^ Ω·cm [268]. High operating temperatures and corrosive conditions are common in aerospace and structural health monitoring, where CNT–SiC composites show promise in piezoelectric or pressure-sensitive applications [269]. The comparative schematic chart summarizes relative enhancements in mechanical, electrical, thermal, and electromagnetic interference (EMI) shielding properties of graphene/CNT nanocomposites across polymer, metal, and ceramic matrices (Figure 9). Furthermore, the anisotropic distribution of graphene or carbon nanotubes (CNTs) in these matrices permits direct conductivity tailoring, providing further flexibility in the integration of functional devices. When taken as a whole, these nanocomposite systems show how high-performance electrical capabilities can be unlocked across a wide range of material platforms through carefully considered filler content, alignment, and interfacial compatibility design.

### 6.4. Tribological Improvements and Wear Resistance

When added to different composite matrices, carbon nanotubes (CNTs) and graphene provide remarkable wear resistance and low-friction characteristics, which have drastically changed the field of tribological engineering. Graphene’s two-dimensional lamellar planes and carbon nanotubes’ tubular morphology are two of their intrinsic structural characteristics that allow them to function as effective solid lubricants. By reducing interfacial shear, dissipating frictional heat, and protecting surfaces from direct asperity contact, these nanofillers create thin, stable transfer layers at sliding interfaces that increase component tribological longevity [270]. Even tiny additions (1–3 weight percent) of graphene or carbon nanotubes (CNTs) significantly increase wear performance in polymer composites, which are extensively employed in mechanical and aerospace systems [271]. For instance, adding graphene to PTFE, nylon, and epoxy matrices has resulted in a notable 60–80% reduction in wear volume loss and a 30–70% decrease in the coefficient of friction (COF). Several synergistic effects are responsible for these improvements [272]. A continuous, lubricious coating that reduces resistance is formed by graphene’s high aspect ratio, which enables it to align along the direction of motion under shear [273]. Furthermore, graphene and CNTs both function as nanoscale load-bearing reinforcements that aid in more evenly distributing mechanical stress throughout the polymer matrix. By preventing direct molecular bonding between opposing surfaces, which is a frequent source of surface degradation in polymers, their presence also reduces adhesive wear [274].

Graphene-enhanced epoxy composites are suitable for high-cycle wear applications since wear experiments by Bazaka et al. show that they maintain their low-friction characteristics even after long sliding cycles [275]. Additionally, transferred graphene films that act as continuous lubricating layers during sliding are confirmed by scanning electron microscopy (SEM) investigations on the counterface [276]. The addition of carbon nanotubes (CNTs) to metal matrix composites (MMCs) has allowed for notable enhancements in wear behavior and friction. For example, under dry sliding, the COF of aluminum supplemented with 2 weight percent CNTs by spark plasma sintering (SPS) decreases from 0.9 to 0.3 [277]. The ability of the CNTs to maintain their structural integrity while sliding, serving as rolling and gliding features that reduce abrasive contact, is responsible for this performance increase. Additionally, CNTs improve the matrix’s resistance to plastic deformation by increasing its hardness and modulus. Because of their strong C–C bonds, which serve as barriers against thermal degradation and oxygen diffusion at the surface, post-wear investigations have shown that CNTs also reduce oxidative wear.

Additionally, graphene has shown remarkable promise in enhancing the tribological properties of ceramic materials. Ceramics like SiO_3_ and AlO_3_ are generally brittle and have low wear resistance when subjected to cyclic loading [278]. But the advent of graphene nanoplates (GNPs) has fundamentally changed this story. According to Wang et al., adding 1.5–2 weight percent graphene to alumina matrices can reduce wear rate by up to 60% and significantly reduce friction under reciprocating sliding [279]. The development of self-lubricating interfacial coatings, which lessen crack start and propagation, makes these advancements possible. Furthermore, by bridging microcracks, graphene decreases grain boundary pull-out and increases interfacial toughness, improving structural reliability and surface integrity [280].

The wear debris from these devices provides more evidence that graphene and carbon nanotubes (CNTs) stabilize the wear process. Raman spectroscopy and focused ion beam (FIB) investigations show that the nanostructures retain their lattice integrity in the face of shear-induced stresses, demonstrating their durability and continued usage as lubricants [281]. Because conventional lubricants fail in high-temperature and high-load applications, this endurance is particularly important. Thus, a paradigm shift in tribological design has been brought about by the incorporation of graphene and carbon nanotubes (CNTs) into polymer, metal, and ceramic matrices. These nanofillers support mechanical strength and thermal stability in addition to lowering wear and friction. For applications where dependability and performance in harsh environments are crucial, such as automotive, aerospace, biomedical, and energy, their multipurpose role makes it possible to design next-generation wear-resistant materials [282].

### 6.5. Corrosion Resistance and Environmental Durability

When applied as protective coatings or reinforcement agents in metal matrices, graphene and carbon nanotubes (CNTs) significantly improve the corrosion resistance and environmental endurance of composite materials. The creation of extremely effective physical barriers is made possible by their special physical and chemical properties, which include high aspect ratios, superior chemical inertness, and impermeability. The main causes of electrochemical corrosion, moisture, oxygen, and chloride ions, are greatly hindered by these nanoparticles. Graphene’s two-dimensional planar structure creates winding diffusion routes in metal matrix coatings that prevent direct contact between the electrolyte and the metal substrate and postpone ion permeation [283]. When graphene is added, electrochemical tests such as potentiodynamic polarization and electrochemical impedance spectroscopy (EIS) have shown a significant decrease in corrosion current densities (Icorr) and a rise in corrosion potential (Ecorr). Rekha et al., for example, demonstrated that Zn–graphene composite coatings on mild steel surfaces showed a ~90% drop in Icorr in 3.5% NaCl solution compared to bare steel, with a corresponding shift in Ecorr of +300 mV, indicating greater thermal stability [284]. These enhancements were ascribed to the homogeneous dispersion and compact layering of graphene, which prevented cathodic and anodic reactions at the metal surface.

CNTs, especially multi-walled carbon nanotubes (MWCNTs), provide corrosion protection by using both electrochemical passivation and physical shielding. CNTs in electrodeposited Ni–CNT coatings improve passivation behavior by promoting the creation of more uniform and adherent oxide layers and enhancing the coating’s mechanical integrity, which makes it more resistant to cracking and mechanical failure [285]. Particularly in acidic or chloride-rich settings, these coatings have shown exceptional resistance to localized corrosion processes such pitting and intergranular assault. Furthermore, when CNTs and graphene are joined, hybrid systems show synergistic benefits, where the combined qualities outperform those of either nanomaterial alone. The barrier properties of graphene and the mechanical reinforcement of carbon nanotubes [286] are advantageous for these systems. These hybrids have excellent coating adherence, great fracture toughness, and even self-healing properties when paired with responsive polymers and integrated in sol–gel matrices or polymeric coatings. In addition to preventing moisture intrusion and surface delamination, these hybrid composites exhibit self-healing microcracks that are induced by variations in pH or temperature. These multifunctional coatings are especially important in industries that need long-term environmental endurance, like biomedical implants, marine structures, and infrastructure subjected to varying environmental stressors [287]. The development of these nanocomposite coatings keeps pushing the limits of both passive and active corrosion mitigation, setting new standards for the creation of robust and intelligent materials.

### 6.6. Process–Structure–Property Correlations

The interplay between fabrication techniques, microstructural features, and final material properties has a significant impact on the performance of CNT- and graphene-based nanocomposites. Functional performance is largely determined by the dispersion of nanofillers, interfacial bonding, and structural integrity of the matrix, particularly in high-performance applications that need electrical conductivity, mechanical resilience, or thermal stability [288]. Achieving homogeneous dispersion of graphene or carbon nanotubes (CNTs) in polymer matrix composites (PMCs) is a major difficulty. The nanofillers are frequently exfoliated and distributed using methods such high-shear mixing, ultrasonication, and three-roll milling [289]. These techniques must be carefully managed, though, because too much energy input during milling or ultrasonication can shorten carbon nanotubes and cause flaws in graphene sheets, decreasing their aspect ratio and weakening the effectiveness of reinforcement. Agglomeration, which is otherwise harmful to the mechanical and electrical properties of the composite, can be reduced and matrix–filler interfacial adhesion enhanced by controlled exfoliation and functionalization, such as carboxylation or amination [290].

Processing techniques such as powder metallurgy (PM), friction stir processing (FSP), and spark plasma sintering (SPS) are common in metallic systems [291]. High-density composites with homogeneous dispersion of nanoscale reinforcements are made possible by SPS’s quick consolidation and low grain growth. Because of the consistent distribution of CNTs and the robust metallurgical bonding, SPS-fabricated Al–CNT composites demonstrated improved tensile strength, thermal stability, and wear resistance [292]. Likewise, FSP permits the in situ incorporation of nanofillers into metal surfaces, resulting in gradient structures that have enhanced thermal conductivity, corrosion resistance, and surface hardness [293]. The brittleness of the matrix and the thermal instability of some nanofillers are the main issues with ceramic matrices [294]. Freeze casting and hot pressing are frequently used to improve densification while preserving microstructural control [295]. In ceramic nanocomposites, interfacial engineering is essential. By adding oxide interlayers or multi-phase hybrid fillers (such as graphene nanoplatelet–SiC–CNT), interfacial mismatch can be reduced, and crack deflection can be improved. demonstrated notable enhancements in heat conductivity and fracture toughness in these systems, underscoring the significance of hybrid filler systems in attaining multifunctionality [296].

Additionally, process optimization is being revolutionized using machine learning (ML) and artificial intelligence (AI) algorithms into materials research. AI can forecast ideal processing parameters, like temperature, pressure, or mixing time, for reaching desired attributes, like maximum conductivity or minimal porosity, by evaluating sizable datasets from simulation and experimental research [297]. Additionally highlighted was the creation of digital twins and ML-enabled predictive modeling platforms that can simulate composite behavior under a variety of processing settings [298]. These technologies play a crucial role in cutting down on development time and directing experimental efforts toward high-performing, application-specific nanocomposites. Thus, the design of next-generation nanocomposite materials continues to be based on the process–structure–property paradigm. In CNT- and graphene-reinforced systems, researchers are achieving previously unheard-of levels of performance and customization through careful control of fabrication processes, real-time structural analysis, and AI-based optimization [299]. For these cutting-edge materials to go from lab research to scalable commercial applications, a comprehensive understanding is essential.

### 6.7. Multiphase Systems and Functional Synergy

As nanocomposites have evolved, single-filler systems have given way to more intricate multiphase hybrid architectures, in which several nano-entities, including metal/metal oxide nanoparticles, carbon nanotubes (CNTs), graphene nanoplatelets (GNPs), and functional polymers, are combined within a single host matrix [300]. To enable unparalleled multifunctionality suited to cutting-edge applications in the aerospace, medicinal, electronics, and energy sectors, this technique seeks to capitalize on synergistic effects resulting from the distinct dimensionality and physical-chemical properties of each component. Typically, hybrid nanocomposites combine two-dimensional (2D) nanosheets like graphene or molybdenum disulfide (MoS_2_), one-dimensional (1D) structures like carbon nanotubes (CNTs), and zero-dimensional (0D) nanoparticles like TiO_2_ and ZnO [301]. A balanced mix of mechanical stiffness, electrical performance, thermal conductivity, and interfacial durability is provided by this multifaceted integration. For example, ternary polymer systems like epoxy–GNP–CNT composites take advantage of the flexibility and crack-bridging capabilities of CNTs, the high planar conductivity and mechanical strength of graphene, and the processability of the polymer matrix [302]. When compared to binary or single-filler composites, these systems may demonstrate improvements in electrical conductivity, mechanical toughness, and thermal stability. Likewise, multiphase combinations like Al–GNP–TiO_2_ have shown simultaneous increases in hardness, wear resistance, and catalytic activity in metal matrix systems [303]. While CNTs provide ductility and dislocation bridging, TiO_2_ nanoparticles provide photochemical or oxidation resistance benefits, while graphene’s lubricity and electronic conductivity lower interfacial friction and facilitate charge transport. These synergistic functions are especially helpful for parts like turbine blades, brake pads, and aerospace structural elements that are subjected to oxidative conditions and mechanical stress [304]. Nevertheless, there are certain difficulties in putting such multiphase systems into practice. Because the different fillers have different surface energy, particle sizes, and aspect ratios, the processing complexity grows exponentially. As the system progresses beyond binary mixtures, it becomes increasingly challenging to achieve homogenous dispersion and prevent agglomeration. Agglomerates frequently serve as locations for defects that impair electrical and mechanical qualities [305].

Surface functionalization techniques are commonly used to lessen these problems. Improved interfacial bonding between the nanofillers and matrix is facilitated using compatibilizers, coupling agents (such as silanes and titanates), and surfactants [306]. Additionally, dispersion quality can be optimized by using sequential mixing strategies, which add different fillers at different stages of the mixing process. For instance, GNP and CNT phases have been distributed independently with little tangling or stacking when three-roll milling and solution mixing are used [307]. A platform for the development of smart materials with electrical conduction, mechanical load bearing, sensing, and catalytic action all within a lightweight and flexible design space is provided by multiphase nanocomposites, which present a promising way to incorporate multiple functionalities into a single material system [308]. Future developments in this field are probably going to depend on the development of scalable processing techniques, predictive modeling for structure–property optimization, and interfacial chemistry.

### 6.8. Environmental and Sustainability Considerations

It is becoming increasingly important to assess the environmental effect, life-cycle performance, and end-of-life sustainability of nanocomposite technologies as they approach widespread commercial implementation. While improved material performance is made possible by the growing usage of engineered nanomaterials like graphene and carbon nanotubes (CNTs), concerns about ecotoxicity, recyclability, and the carbon footprint of their production and disposal are also raised [309]. Because of their inherent stability and lack of biodegradability, graphene and carbon nanotubes (CNTs) pose questions regarding environmental persistence and bioaccumulation in terrestrial and aquatic ecosystems [310]. At high concentrations, these substances can cause oxidative stress and membrane damage in specific cell types and microorganisms. An increasing movement to create environmentally safe nanocomposite materials with biodegradable polymers and biofunctionalized fillers has resulted from this. To create environmentally safe systems for biomedical devices, packaging, and agricultural sensors, matrices like polylactic acid (PLA), polycaprolactone (PCL), and starch-based biopolymers are being investigated in combination with surface-modified carbon nanotubes (CNTs) or green graphene (produced via electrochemical or mechanical exfoliation) [311]. Life-cycle assessments, or LCAs, are becoming a crucial tool for comprehending the effects of nanocomposite materials from birth to death. For example, powerful oxidants (such as KMnO_4_ and H_2_SO_4_) used in the conventional Hummers technique for graphene synthesis produce emissions and chemical waste. On the other hand, environmentally friendly synthesis methods such liquid-phase exfoliation in safe solvents, biomass-derived carbonization, and electrochemical exfoliation have significantly smaller environmental footprints [312]. Chemical vapor deposition (CVD) methods employing renewable carbon sources provide a more scalable and cleaner pathway, however technical optimization is still under progress [313]. under a similar vein, CNTs produced by arc discharge or laser ablation require significant energy input and metal catalysts.

Circular design and recyclability are important factors. Because of filler–matrix bonding and thermal breakdown behavior, graphene or carbon nanotube-reinforced polymer nanocomposite materials are generally not thermodynamically compatible with traditional plastic recycling processes [314]. Nevertheless, advancements in low-temperature reprocessing and additive manufacturing (AM) are starting to remove these obstacles. For instance, AM makes it possible to fabricate functional parts in near-net shape with little waste and closed-loop recycling methods that use thermal recovery or selective depolymerization are being developed for the recovery of nanocomposite materials [315]. Additionally, the incorporation of sensor-embedded functions and self-healing mechanisms might prolong the service life of nanocomposite-based components, thereby lowering the overall environmental load and the requirement for frequent replacement [316]. The adoption of thin, multifunctional coatings with smart deterioration profiles in applications such as printed antennas, structural health monitoring, and EMI shielding is contributing to the field’s shift towards sustainable engineering methods [317]. Therefore, the next generation of nanocomposites now incorporates sustainability and environmental factors. The benefits of CNTs and graphene can be utilized while adhering to international sustainability objectives through ethical material selection, green synthesis, and circular economy techniques. Realizing the full potential of nanocomposites in a world where resources are limited will need the convergence of material science, environmental engineering, and regulatory frameworks.

## 7. Unifying Mechanisms Governing Graphene/CNT Nanocomposite Performance Across Matrices

**Figure 10:**
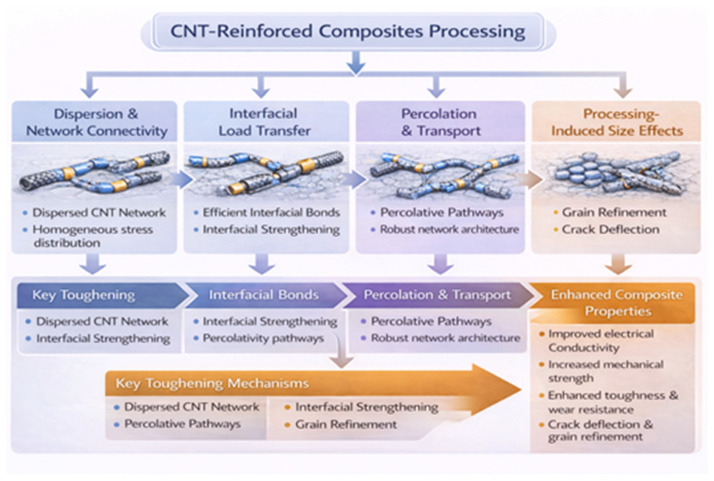
Unified mechanistic framework illustrating dominant reinforcement and transport mechanisms in graphene/CNT nanocomposites across polymer, metal, and ceramic matrices: influence of nanofiller dispersion on network connectivity and stress distribution; matrix-specific interfacial load-transfer mechanisms in polymers, metals, and ceramics; percolation behavior in hybrid graphene–CNT systems leading to reduced percolation thresholds and stabilized conductive pathways; processing-induced size effects linking fabrication routes to microstructural refinement and multifunctional performance enhancement.

Although graphene/CNT nanocomposites are often discussed separately for polymer, metal, and ceramic matrices, a comparative and mechanism-driven analysis reveals that their performance is governed by a common set of underlying parameters, largely independent of matrix chemistry. Figure 10 presents a unified mechanistic interpretation of graphene/CNT reinforcement across polymer, metal, and ceramic matrices by correlating dispersion state, interfacial load transfer, percolation behavior, and processing-induced size effects with macroscopic property enhancement [318]. As illustrated in Figure 10, uniform dispersion of graphene platelets and carbon nanotubes enables the formation of interconnected networks that homogenize stress distribution and establish continuous electrical and thermal transport pathways, whereas agglomeration introduces stress concentrators, disrupts percolation, and limits effective reinforcement across all matrix systems. Load transfer mechanisms, are governed by matrix-specific interfacial interactions: interfacial shear and chain entanglement dominate polymer nanocomposites, metallurgical bonding and diffusion-controlled interfaces regulate metal matrix systems, and mechanical interlocking combined with localized chemical bonding at grain boundaries controls reinforcement in ceramics, where crack bridging and deflection are prevalent. Despite these differences, interfacial integrity and nanofiller geometry remain universal determinants of reinforcement efficiency. The role of percolative transport is highlighted, where hybrid graphene–CNT architectures reduce percolation thresholds and stabilize conductive networks by allowing CNTs to bridge adjacent graphene sheets, minimize tunneling distances, and maintain transport continuity under mechanical deformation, resulting in superior electrical, thermal, and sensing performance compared to single-filler systems. Finally, Figure 10 summarizes the influence of processing-induced size effects, demonstrating how advanced processing routes refine grains, confine polymer chains, and control nanofiller orientation, thereby enhancing strength, stiffness, and fracture resistance. In metals and ceramics, grain refinement and dislocation pinning dominate strengthening mechanisms, while in polymers, chain confinement and interfacial stress transfer govern mechanical response. Additionally, nanoscale fillers increase crack path tortuosity and promote energy dissipation through pull-out and interfacial sliding.

Dispersion quality represents the most critical and universal parameter influencing performance [319]. In polymer matrices, homogeneous dispersion of graphene and CNTs enables the formation of interconnected conductive networks at low filler loadings, reducing percolation thresholds and enhancing electrical and thermal transport [320]. In metal and ceramic matrices, uniform dispersion governs grain refinement, defect pinning, and crack-deflection efficiency by restricting matrix grain growth and promoting homogeneous stress distribution. Despite differences in processing routes solution blending, melt mixing, powder metallurgy, or sintering agglomeration consistently leads to premature failure, poor reproducibility, and diminished multifunctional performance [321]. This convergence across matrices highlights dispersion as a universal bottleneck rather than a matrix-specific challenge, underscoring the importance of dispersion-control strategies that are transferable across material systems.

Interfacial load transfer emerges as the second unifying mechanism. In polymer nanocomposites, stress transfer is mediated by van der Waals interactions, covalent functionalization, or polymer chain wrapping at the graphene/CNT interface [322]. In metal matrices, load transfer depends on metallurgical bonding, interfacial wetting, and suppression of brittle carbide formation, while in ceramics, crack bridging, pull-out, and grain-boundary anchoring dominate reinforcement mechanisms [323]. Despite these apparent differences, the efficiency of load transfer is fundamentally governed by interfacial shear strength, effective contact area, and nanofiller geometry. High-aspect-ratio CNTs facilitate stress bridging across microcracks, while graphene platelets provide large interfacial contact areas for stress redistribution [324]. Hybrid graphene–CNT architectures exploit both effects simultaneously, leading to more efficient load transfer than either filler alone. Thus, interfacial mechanics provides a common framework for understanding reinforcement across polymeric, metallic, and ceramic systems. Percolation behavior constitutes a third universal mechanism controlling functional properties [325]. Electrical and thermal conductivity enhancements in graphene/CNT nanocomposites consistently exhibit sharp threshold behavior once an interconnected filler network is established. This phenomenon is observed across polymers, retained in metal matrices after consolidation, and increasingly exploited in ceramic systems for multifunctional applications such as sensing and electromagnetic interference (EMI) shielding [326]. Hybrid graphene–CNT systems consistently demonstrate lower percolation thresholds than single-filler composites due to their complementary one-dimensional and two-dimensional geometries. CNTs act as conductive bridges between graphene platelets, reducing inter-sheet contact resistance and enabling robust network formation at reduced filler contents. This hybrid percolation mechanism explains why significant conductivity enhancements are often achieved at relatively low graphene/CNT loadings across diverse matrices [327].

Processing-induced size effects provide a fourth unifying mechanism linking processing, microstructure, and macroscopic performance. In metals consolidated by spark plasma sintering or hot pressing, graphene and CNTs suppress grain growth, leading to nanoscale grain refinement and Hall–Petch strengthening. In polymer matrices, nanoconfinement of polymer chains near graphene/CNT interfaces restricts chain mobility, enhancing modulus and thermal stability [328]. In ceramic systems, nanocarbon fillers inhibit grain coarsening and deflect crack propagation paths, improving fracture toughness. Importantly, these size effects frequently act synergistically with nanofiller reinforcement, explaining why relatively low graphene/CNT contents can yield disproportionate property gains. This synergy further emphasizes that processing routes are not merely fabrication steps but active contributors to performance through microstructural control. Therefore, these observations indicate that graphene/CNT nanocomposites should be designed using a mechanism-driven framework rather than matrix-specific heuristics [329]. By focusing on dispersion control, interfacial mechanics, percolation architecture, and size effects, researchers can rationally optimize processing routes, filler geometry, and surface chemistry across polymers, metals, and ceramics. Such a unified design philosophy not only enhances predictive capability but also improves reproducibility and scalability, which are essential for the successful translation of graphene/CNT nanocomposites from laboratory studies to industrial applications.

**Figure 11:**
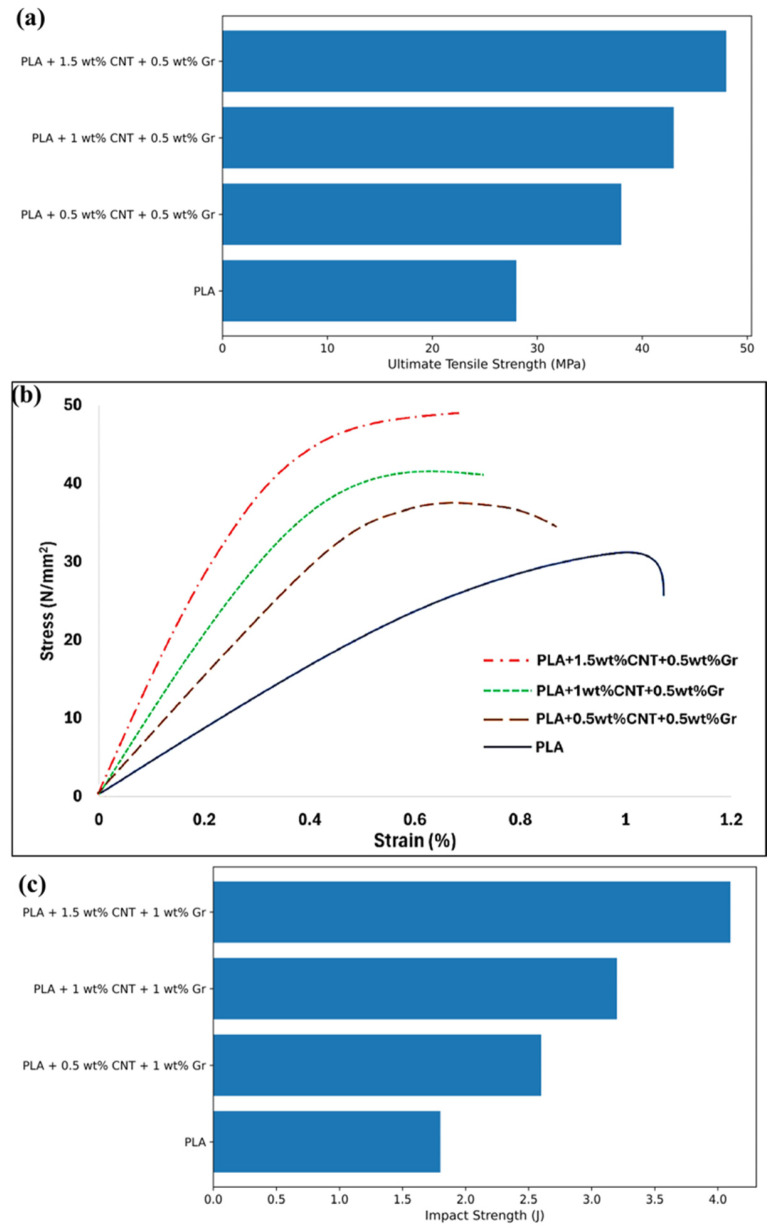
Representative experimental mechanical properties of hybrid graphene/CNT-reinforced PLA nanocomposites (**a**) ultimate tensile strength as a function of hybrid filler composition, (**b**) stress-strain behavior highlighting stiffnessa nd strength enhancement and (**c**) impact strength variation. The data illustrates the non-linear dependence of mechanical performance on filler loading, reflecting the combined effects of dispersion quality, interfacial load transfer, and network formation [330].

The experimental results drawn from the literature provide quantitative validation of the processing–structure–property relationships discussed throughout this review. As illustrated in Figure 11, hybrid graphene/CNT reinforcement leads to a pronounced enhancement in the mechanical performance of PLA-based nanocomposites. The ultimate tensile strength data (Figure 11a) show a systematic increase with increasing CNT content at a fixed graphene loading, reaching a maximum for the PLA + 1.5 wt.% CNT + 0.5 wt.% Gr composition. This trend reflects efficient stress transfer and the formation of a well-connected hybrid filler network at low-to-moderate loadings. The stress–strain curves (Figure 11b) confirm this behavior, with hybrid-filled systems exhibiting higher stiffness and ultimate strength than neat PLA, while excessive filler addition begins to reduce strain-to-failure due to matrix constraint and stress localization. Impact strength results (Figure 11c) highlight the multifunctional trade-offs inherent to hybrid graphene/CNT systems [330]. While impact resistance improves with increasing hybrid filler content attributed to crack deflection, crack pinning, and enhanced energy dissipation—the ductility of the matrix is progressively reduced. These results clearly indicate the existence of an optimal reinforcement window in which strength and toughness are simultaneously improved without excessive loss of deformability [331].

**Figure 12:**
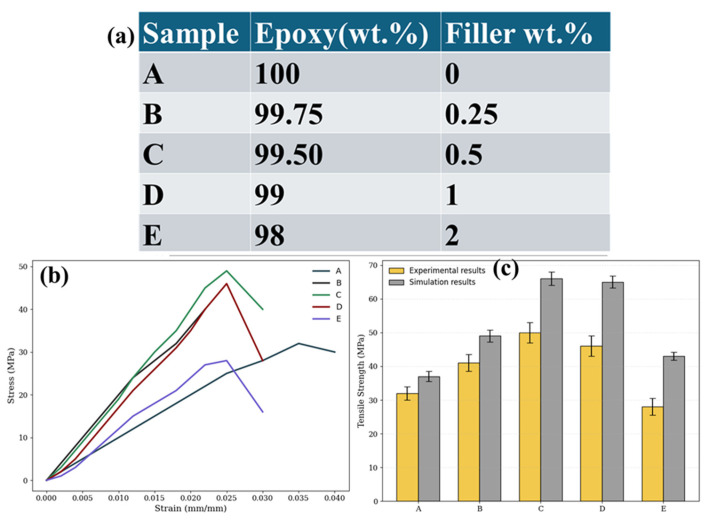
Representative experimental and numerical mechanical response of hybrid graphene/CNT-reinforced epoxy composites (**a**) sample notation, (**b**) stress-strain behavior of samples A–E with increasing hybrid filler content, and (**c**) comparison between experimentally measured and simulated tensile strength. The results illustrate non-linear strengthening behavior, good experiment–simulation agreement, and the existence of an optimal hybrid filler loading governed by dispersion quality and interfacial load transfer.

**Figure 13:**
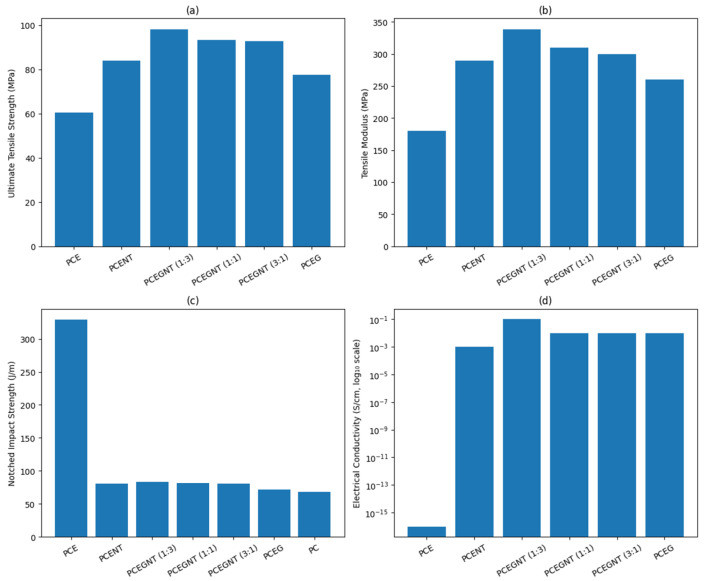
Mechanical and electrical properties of hybrid graphene/CNT-reinforced epoxy composites (**a**) ultimate tensile strength, (**b**) tensile modulus, (**c**) notched impact strength, and (**d**) electrical conductivity for different graphene/CNT ratios. The data highlight synergistic strengthening and conductivity enhancement in hybrid systems, alongside reduced impact resistance at higher filler loadings due to increased matrix stiffening and network densification.

Complementary evidence is provided by epoxy-based hybrid nanocomposites shown in Figure 12a,b. Stress–strain responses for samples A–E (Figure 12b) demonstrate a steady increase in stiffness and tensile strength from the neat epoxy (A) to intermediate hybrid compositions (B–C), followed by reduced ductility at higher filler contents (D–E). The comparison between experimental and simulated tensile strength (Figure 12c) shows good agreement, confirming that the observed strengthening trends are governed by dispersion quality and interfacial load-transfer efficiency rather than experimental variability alone [332]. Further validation of multifunctional behavior is observed in Figure 13, which summarizes tensile strength, tensile modulus, impact strength, and electrical conductivity for epoxy-based graphene/CNT hybrids. The PCEGNT (1:3) composition exhibits the highest tensile strength and modulus (Figure 13a,b), indicating effective hybrid synergy and network formation. However, impact strength (Figure 13c) decreases relative to the neat polymer, revealing increased brittleness at higher nanofiller loadings. In contrast, electrical conductivity (Figure 13d) increases by several orders of magnitude upon hybrid filler addition, confirming the establishment of percolated conductive pathways [333]. Collectively, these experimental findings demonstrate that hybrid graphene/CNT nanocomposites across polymer and thermoset matrices exhibit non-linear, mechanism-governed property evolution. The results strongly support the central thesis of this review: optimal performance is achieved through balanced dispersion, interfacial bonding, and percolation, rather than through indiscriminate increases in nanofiller content [334]

## 8. Conclusions and Future Outlook

The development of carbon-based nanocomposites has followed a distinct and instructive trajectory over the past three decades. The discovery of carbon nanotubes in 1991 marked the first breakthrough, revealing the extraordinary mechanical strength, electrical conductivity, and high aspect ratio of one-dimensional carbon nanostructures. Subsequent research throughout the 1990s and early 2000s focused primarily on CNT synthesis, dispersion, and reinforcement mechanisms in polymer and metal matrices, establishing foundational concepts such as percolation, load transfer, and interfacial stress mediation. The isolation of graphene in 2004 introduced a complementary two-dimensional building block with exceptional surface area, thermal conductivity, and barrier properties. Initial efforts emphasized fundamental characterization and single-filler composite systems, while the period following 2010 witnessed a decisive shift toward hybrid graphene/CNT architectures. This transition reflected a growing recognition that combining one- and two-dimensional nanocarbons enables synergistic networks that outperform individual fillers, particularly in terms of multifunctionality and percolation efficiency.

Despite substantial progress, the translation of graphene/CNT nanocomposites from laboratory demonstrations to industrially viable materials remain incomplete. Persistent challenges include achieving reproducible large-scale dispersion, controlling interfacial chemistry without degrading intrinsic nanocarbon properties, and balancing cost, quality, and throughput in synthesis routes. Variability arising from dopants, residual catalysts, and processing-induced defects continues to limit consistency across studies and applications. Moreover, while high-performance enhancements are frequently reported, systematic understanding of size effects, long-term durability, environmental stability, and life-cycle impact is still lacking. In metal and ceramic matrix systems, interfacial reactions and thermal instability impose additional constraints, whereas polymer-based composites must reconcile processability with mechanical robustness and functional reliability. These unresolved issues underscore the need for standardized processing protocols, quantitative structure–property correlations, and realistic benchmarking against application-specific requirements.

Looking forward, the future of graphene/CNT nanocomposites will be shaped by integration rather than isolated material optimization. Advances in scalable synthesis—such as atmospheric-pressure and roll-to-roll CVD—combined with low-temperature consolidation techniques offer promising routes toward economically viable production. Modeling-guided design, supported by density functional theory, molecular dynamics, and emerging machine-learning frameworks, is expected to play a central role in predicting dopant effects, dispersion behavior, and interfacial performance, thereby reducing empirical trial-and-error. Equally important will be the adoption of application-driven design philosophies that prioritize manufacturability, sustainability, and functional reliability alongside peak performance. By aligning materials innovation with realistic processing constraints and end-use requirements, graphene/CNT hybrid nanocomposites are well positioned to transition from advanced research materials to practical components in structural, electronic, energy, and protective systems.

## Figures and Tables

**Table 1:** Summary of Key Literature on Graphene and CNT-Reinforced Nanocomposites.

Reference	Matrix	Filler System	Processing Route	Key Property Outcomes
Zhang et al. [235]	PMMA	GNP + MWCNT	Solution blending	Reduced percolation threshold; synergistic enhancement of mechanical and electrical properties
Bressanin et al. [236]	PMMA	MWCNT	In situ polymerization	Conductivity ≈ 10 S m^−1^ at 2.2 wt.%; improved thermal stability
Zarei et al. [237]	Cu	CNT/Graphene	Mechanical milling + SPS	Grain refinement (20–36 nm); density 95–97%; improved hardness and yield strength
Munir et al. [238]	Ti	MWCNT	HEBM + SPS	Improved densification and hardness; partial in situ TiC formation
Azarniya et al. [239]	Ceramics	CNT	SPS	Enhanced fracture toughness and electrical conductivity
Halder et al. [240]	Al_2_O_3_	MWCNT	Gel combustion + SPS	Increased hardness; significant wear reduction
Sushmita et al. [241]	Polycarbonate	Doped graphene + CNT	Solution blending	EMI shielding up to −33 dB via magnetic/dielectric losses
Anas et al. [242]	Al–Cu–Mg alloy	CNT/Ni-coated CNT	Mechanical milling + extrusion	Grain refinement; strength enhancement with reduced ductility

## Data Availability

The original contributions presented in this study are included in the article. Further inquiries can be directed to the corresponding authors.
